# Cinnamaldehyde triggers cell wall remodeling and enhances macrophage-mediated phagocytic clearance of *Candida albicans*

**DOI:** 10.3389/fcimb.2025.1647320

**Published:** 2025-09-15

**Authors:** Zhaoling Shi, Jiajia Lin, Wenqian Li, Feng Chen, Wenna Zhang, Yue Yang, Kelong Ma

**Affiliations:** ^1^ College of Integrated Chinese and Western Medicine (College of Life Science), Anhui University of Chinese Medicine, Hefei, China; ^2^ Institute of Integrated Chinese and Western Medicine, Anhui Academy of Chinese Medicine, Hefei, China; ^3^ Anhui Province Key Laboratory of Chinese Medical Formula, Anhui University of Chinese Medicine, Hefei, China

**Keywords:** *Candida albicans*, cell wall, cinnamon, macrophage, phagocytic clearance

## Abstract

**Introduction:**

*Cinnamomum cassia*, a traditional Chinese medicinal herb, possesses cinnamaldehyde (CIN) with well-documented antifungal and immunomodulatory properties. Although CIN inhibits *Candida albicans* (*C. albicans*) growth, its role in macrophage-mediated clearance remains poorly understood.

**Methods:**

Here, we evaluated CIN's antifungal activity using MIC determination, spot assays, and time-growth curves. Cell wall disruption (β-glucan and chitin exposure) was assessed by transmission electron microscopy (TEM), confocal laser scanning microscopy (CLSM), and flow cytometry.

**Results:**

Transcriptomic and functional enrichment analyses revealed that CIN compromises cell wall integrity by altering 123 differentially expressed genes (DEGs), particularly those governing hyphal development, cell wall biosynthesis, and biofilm formation. Specifically, CIN downregulated genes associated with β-glucan exposure, mannosylation, and chitin synthesis, and upregulated components of the Cek1/MAPK pathway. CIN-enhanced macrophage phagocytosis significantly increased fungal clearance and reduced fungal escape, as shown by flow cytometry, propidium iodide staining, and lactate dehydrogenase release assays. CIN-pretreated fungi activated the Dectin-1/Syk/CARD9/NF-κB cascade, leading to elevated pro-inflammatory cytokine secretion.

**Discussion:**

Mechanistically, CIN induces β-1,3-glucan exposure on *C. albicans*, thereby promoting Dectin-1-mediated phagocytosis and clearance. These findings provide an experimental basis for developing CIN as a novel antifungal therapeutic.

## 1 Introduction

*C. albicans* is a commensal and pathogenic fungus that commonly colonizes the oral cavity, gastrointestinal tract, and urogenital system of healthy individuals (Wang et al., 2019). In immunocompetent hosts it maintains microbial equilibrium, but under immunosuppression or microbiome dysbiosis it becomes to pathogenic, causing infections ranging from superficial candidiasis to life-threatening systemic disease (Peroumal et al., 2022; Zhao et al., 2022). Current antifungal agents targeting membrane sterols (azoles/polyenes) or β-1,3-glucan synthesis (echinocandins) face declining efficacy due to emerging resistance. Rising azole resistance in *C. albicans* and pan-drug-resistant *Candida auris* strains–driven by both clinical overuse and environmental selection pressure–has become a major concern (Pristov and Ghannoum, 2019). Thus, developing novel antifungal agents and combination therapies is essential for overcoming resistance in invasive fungal infections (Fuentefria et al., 2018).

The fungal cell wall is the primary interface in host–pathogen interactions, functioning as both a structural barrier and a reservoir of immunostimulatory pathogen–associated molecular patterns (PAMPs) such as β-glucans and chitin. These PAMPs are recognized by host pattern-recognition receptors (PRRs), triggering defensive immune responses (De Assis et al., 2022). The wall’s architecture, comprising diverse polysaccharides and glycoproteins, directly dictates fungal virulence and immune evasion (Huo et al., 2025). *C. albicans* dynamically remodels its wall in response to environmental stresses, masking immunogenic epitopes while exposing β-glucans during immune attack to modulate host responses (Hopke et al., 2016). This remodeling regulates PAMP availability and strongly influences inflammatory outcomes (Esher et al., 2018; Li et al., 2024). Clinically relevant stressors such as caspofungin, acidic pH, and oxidative conditions compromise wall integrity, which exerts dual antimicrobial effects: direct fungicidal damage and enhanced immune recognition of exposed PAMPs (Sah et al., 2023).

Cinnamaldehyde (CIN), a bioactive constituent of *Cinnamomum cassia*, has attracted considerable attention for its antifungal properties. Studies demonstrate efficacy against multiple fungal pathogens, highlighting its therapeutic potential. CIN exerts multifaceted antifungal effects through cell structure disruption, metabolic interference, and induction of fungal apoptosis (Chen et al., 2019a; Huang et al., 2019). Our previous study revealed that CIN alleviates *C. albicans* infection in murine ulcerative colitis models by inducing surface β-1,3-glucan exposure (Ma et al., 2020). Nevertheless, the precise mechanisms through which CIN remodels the fungal cell wall and modulates subsequent immune recognition remain elusive.

The present study demonstrates that CIN strengthens host defense against *C. albicans* by disrupting fungal cell wall integrity to expose immunogenic β-1,3-glucan, thereby enhancing macrophage phagocytosis, and reducing fungal escape. Transcriptomic profiling and molecular docking further suggest key molecular targets underlying these effects. Our findings provide compelling experimental evidence for CIN as an immune-enhancing antifungal candidate.

## 2 Materials and methods

### 2.1 Cell lines, strains, and culture

THP-1 human macrophage and RAW 264.7 murine macrophage-like cell lines were sourced from the National Collection of Authenticated Cell Cultures (Shanghai, China). Cells were maintained in RPMI-1640 medium (for THP-1) or Dulbecco’s Modified Eagle Medium (for RAW 264.7), each supplemented with 10% (v/v) fetal bovine serum, and incubated at 37°C in a humidified incubator with 5% CO_2_. THP-1 cells were exposed to 100 ng/mL phorbol 12-myristate 13-acetate (PMA) (P8139, Sigma-Aldrich, USA) for 24 h to differentiate into macrophage-like cells. The identification of THP-1 macrophages is described in **Supplementary Figure S1**. The *C. albicans* strain SC5314 was supplied by Professor Yuanying Jiang from the Naval Medical University of China. *C. albicans* strain was grown in liquid YPD medium (HB5193-1, Hope Biotechnology, China) at 37°C to reach the exponential phase, then centrifuged at 800 × *g* and 4°C, and resuspended in phosphate-buffered saline (PBS, pH 7.0) at a concentration of 2.0 × 10^8^ colony-forming units (CFUs)/mL.

### 2.2 Antifungal assay

The MIC of CIN against *C. albicans* SC5314 was determined by the broth dilution method in 96-well plates, following the CLSI M27-A3 guidelines (Montoya et al., 2020; Pan et al., 2024). Briefly, two-fold serial dilutions of CIN (1.953 to 125 μg/mL) in RPMI 1640 medium (Gibco, 31800022) were dispensed into 96-well plates. A yeast inoculum (1×10^3^ CFU/mL) was added and incubated at 37°C for 24 h. The MIC was defined as the lowest drug concentration that inhibits visible cell growth.

### 2.3 Cell viability

*C. albicans* SC5314 viability was assessed by mitochondrial dehydrogenase activity using the Cell Counting Kit-8 (CCK-8; Biosharp Life Sciences, BS350B) (Jin et al., 2021). Yeast cells (2.0×10^6^ CFU/mL in RPMI 1640) were incubated with two-fold serially diluted CIN (7.8125 to 250 μg/mL) in 96-well plates at 37°C for 12 h. Next, 20 μL of CCK-8 reagent was added to each well and incubated at 37°C for 30 min. Absorbance was measured at 450 nm for each well using a K3 microplate reader (Thermo Scientific, USA).

### 2.4 Time-growth curve

Time-growth curves were assessed according to CLSI M26-A guidelines. *C. albicans* SC5314 in the logarithmic phase was adjusted to 5 × 10^5^ CFU/mL in RPMI 1640 (Gibco, 31800022) and incubated with CIN (15.625 to 62.5 μg/mL) or caspofungin (0.0039 μg/mL, Shanghai Yuanye Bio-Technology Co., Ltd., 179463-17-3) at 37°C with shaking (180 rpm). Aliquots were collected at 0, 4, 8, 12, 16, and 24 h, serially diluted in sterile PBS, plated on YPD agar (HB5193, Hope Biotechnology, China), and incubated at 37°C for 24 h. Viable colonies were enumerated and expressed as mean log^10^ CFU/mL ± SD (n = 3 biological replicates).

### 2.5 Spot assay

*C. albicans* SC5314 susceptibility to CIN was evaluated using a modified CLSI M44-A2 spot assay (Sharma et al., 2020). Logarithmic-phase yeast cells were serially diluted 10-fold in sterile PBS to concentrations of 1×10^2^ to 1×10^6^ CFU/mL. Then, the cells were incubated with CIN (15.625, 31.25, 62.50 μg/mL), caspofungin (0.0039 μg/mL, positive control), PRIM1640 (negative control) in a 1:1 ratio at 37°C for 24 h. A 5 μL volume of the co-incubation solution was spotted onto YPD agar. Plates were incubated at 37°C for 24 h. Growth inhibition was quantified by comparing colony density gradients to controls.

### 2.6 TEM

*C. albicans* SC5314 cells were pelleted (800 × *g*, 5 min) and fixed with 2.5% glutaraldehyde (G916054, Macklin, China) in 0.1 M sodium cacodylate buffer (pH 7.4) for 12 h at 4°C, and post-fixed with 1% osmium tetroxide (OsO_4_) for 1.5 h. Then, the samples were dehydrated through an ethanol gradient (30%→100%), infiltrated with Spurr’s epoxy resin, and polymerized at 60°C for 48 h. Ultrathin slices (70 nm) were cut using a Leica UC7 ultramicrotome, stained with 1% uranyl acetate (15 min), and Reynolds’ lead citrate (5 min), then examined using a HT7700 TEM at 80 kV (Hitachi, Japan). Morphological alterations were imaged at 10,000–50,000× magnification.

### 2.7 CLSM and flow cytometric analysis

Surface exposure of β-1,3-glucan was detected using a modified immunofluorescence method (Guirao-Abad et al., 2018; Wagner et al., 2023). *C. albicans* SC5314 cells were blocked with 3% BSA/PBS (w/v) for 1 h at 25°C, incubated with anti-β-1,3-glucan monoclonal antibody (Clone 400-2, Biosupplies, Australia, 1:200 dilution) at 37°C for 2 h, then labeled with Cy3-conjugated goat anti-mouse IgG (A22210, Abbkine, 1:500) for 1 h in the dark. For chitin detection, cells were stained with 10 μg/mL Calcofluor White (CFW; Sigma-Aldrich, 18909) in PBS for 30 min at 4°C with gentle shaking. After PBS-Tween washes, cells were affixed onto poly-L-lysine-coated slides. Confocal imaging was performed on a Stellaris 5 (60× oil objective; excitation/emission: Cy3 552/570 nm, CFW 405/433 nm). The fluorescence intensity was quantified using a BD FACSCelesta™ flow cytometer (BD Biosciences). CFW/β-glucan fluorescence intensity was analyzed using ImageJ v1.53 (NIH) with rolling ball background subtraction, and population statistics were processed with FlowJo v10.8.1 (BD Biosciences).

### 2.8 RNA isolation and sequencing

*C. albicans* SC5314 cells (2×10^5^ CFU/mL) were cultured in RPMI-1640 medium supplemented with 10% FBS at 37°C for 24 h, with or without CIN (31.25 μg/mL). Total RNA was extracted using the MJZol kit (Majorbio, Shanghai, China) according to the manufacturer’s protocol. The RNA quality was evaluated with the 5300 Bioanalyzer (Agilent, USA) and quantified using the ND-2000 (NanoDrop Technologies). Poly(A)-enriched mRNA was isolated with oligo(dT) beads and then converted to cDNA using a SuperScript kit (Invitrogen, CA, USA). The cDNA library was amplified with 15 PCR cycles using Phusion DNA polymerase and sequenced on an Illumina NovaSeq 6000 system (Majorbio, Shanghai, China). Raw and processed gene expression data are accessible in the GEO database under accession number GSE262904.

### 2.9 Transcriptomic analysis

Raw reads were trimmed and quality-checked with fastp v0.23.2 (Chen et al., 2018). Clean reads were aligned to the *C. albicans* SC5314 reference genome (GCF_000182965.3) using HISAT2 (Kim et al., 2015). Transcript assemblies were generated using StringTie v2.2.4 (Pertea et al., 2015) in reference-guided mode. Gene expression quantification was performed with RSEM v1.3.3 (Li and Dewey, 2011), with abundance reported in Transcripts Per Million (TPM). Differentially expressed genes (DEGs) were identified using DESeq2 v1.38.3 with an absolute log_2_ fold change >1 and a false discovery rate (FDR) <0.05. Gene Ontology (GO) functional enrichment and Kyoto Encyclopedia of Genes and Genomes (KEGG) pathway analyses were performed using Goatools and KOBAS, respectively (Xie et al., 2011). Significance was defined as an FDR-corrected p-value ≤ 0.05 after multiple testing adjustment.

### 2.10 RT-qPCR

RNA was extracted from *C. albicans* SC5314 using the Yeast RNA kit (AC0501, SparkJade, China) and from THP-1 and RAW264.7 cells using the FastPure kit (RC112-01, Vazyme, China), following the manufacturer’s guidelines. RNA concentrations were assessed with a NanoDrop One (Thermo Scientific, USA). cDNA synthesis utilized 1 μg of total RNA with HiScript III RT SuperMix (Vazyme R323-01) under the following conditions: 42°C for 2 min to remove residual genomic DNA→55°C for 15 min→85°C for 5 s. RT-qPCR primers (**Supplementary Table S1**) amplified targets using SYBR Green Master Mix (11184ES08, YEASEN) on a LightCycler 96 (Roche): 95°C for 2 min → [95°C for 10 s → 60°C for 30 s] × 40 cycles → melt curve 65 to 95°C. *ACT1* (*C. albicans* SC5314) and *ACTB* (THP-1 and RAW264.7) served as internal reference genes for data normalization, and the 2-ΔΔCT method was used for relative quantification.

### 2.11 Molecular docking

Molecular docking simulations were performed to investigate interactions between CIN and 31 differentially expressed proteins implicated in fungal cell wall dynamics. The 3D structure of CIN (CID 637511) was obtained from the PubChem database and optimized using the MMFF94 force field in Open Babel v3.1.1. Target protein structures were obtained from AlphaFold DB (v2.3.2) with predicted local distance difference test (pLDDT) scores that exceeded 85. The docking study was performed using a semi-flexible docking approach with AutoDock Vina (version 4.2.6). Docking conformations were generated and analyzed for the lowest binding energy using PyMOL (v.2.5.4).

### 2.12 Macrophage phagocytosis assay

Fluorescence microscopy: RAW264.7 cells (6×10^5^ cells/well) and THP-1 cells (1.6×10^6^ cells/well) were seeded onto confocal plates and incubated overnight. *C. albicans* SC5314 (pre-treated ± 31.25 μg/mL CIN, 37°C for 12 h) was labeled with 10 μg/mL Calcofluor White-V450 (18909, Sigma-Aldrich, USA) for 10 min. Fungi and macrophages were co-cultured at multiplicity of infection (MOI) of 3:1 in RPMI-1640 for 1 or 3 h at 37°C. Non-internalized fungi were removed by three PBS washes. Cells were fixed with 4% paraformaldehyde (PFA; Beyotime P0099) and imaged using a Stellaris 5 confocal microscope (Leica, Germany). Phagocytic index = (Number of internalized fungi/Total macrophages) × 100 from ≥ 3 fields per group (Tripathi et al., 2020).

Flow cytometric analysis: Macrophages were co-cultured with CFW-labeled *C. albicans* (MOI 3:1) for 1 or 3 h. Cells were collected, washed with cold PBS, and stained with PE Anti-Mouse/Human CD11b Antibody (E-AB-F1081D, Elabscience) for 30 min at 4°C. Fluorescence density was measured using a BD FACS CelestaTM flow cytometer (BD Biosciences). Phagocytic rate (%) = [Number of macrophages phagocytosing *C. albicans* (CFW^+^CD11b^+^)/Total number of macrophages (CFW^+^CD11b^+^ + CD11b^+^)] × 100%.

### 2.13 Macrophage-mediated clearance assay of *C. albicans*

*C. albicans* SC5314 (1×10^6^ CFU/mL) was exposed to CIN (31.25 μg/mL) at 37°C for 12 h. The CIN-treated fungi were cocultured with RAW264.7 or PMA-differentiated THP-1 macrophages at an MOI of 3 in 96-well plates. After incubation (37°C, 1 or 3 h), supernatants were collected for XTT reduction assay to assess residual fungal metabolic activity. Fungal survival (%) was calculated as the ratio between OD_treated_ and OD_untreated_ at 492 nm. OD_treated_ represents the OD value of *C. albicans* treated with CIN and/or macrophages, while OD_untreated_ is set as OD value of *C. albicans* with no treatment. For intracellular fungal quantification, macrophages were washed twice with PBS and lysed with 0.5 mL of 2% (w/v) sodium dodecyl sulfate (SDS; Sigma-Aldrich) in PBS (15 min, 37°C). Lysates were serially diluted (10-fold in PBS), plated on YPD agar (HB5193, Hope Biotechnology, China), and incubated (37°C, 24 h). Colony-forming units (CFUs) were enumerated and normalized to macrophage-free *C. albicans* controls.

### 2.14 Lactate dehydrogenase cytotoxicity assay

RAW264.7 macrophages and PMA-differentiated THP-1 cells were cultured in 96-well plates and infected with *C. albicans* SC5314 at an MOI of 3 for 6, 10, 12, and 14 h. Supernatants (120 μL/well) were collected and centrifuged to remove debris. LDH release was quantified using an LDH Assay Kit (C0017, Beyotime Biotechnology, China) according to the instructions. Absorbance was measured at 490 nm. The relative release of LDH was calculated as the percentage of LDH activity in the cell culture supernatants compared to the total LDH activity in both the media and the cells.

### 2.15 Propidium iodide staining of macrophages

PMA-differentiated THP-1 macrophages were seeded in 96-well plates and cultured overnight. Cells were infected with *C. albicans* SC5314 pre-treated with or without CIN (31.25 and 62.5 μg/mL) at an MOI of 3, followed by the addition of 500 ng/mL PI (Sparkjade, China). Fluorescence microscopy was used to visualize and capture PI positive cells at 1, 4, 8, and 12 h post-infection, with observations carried out in a minimum of three independent fields of view. Macrophage counts in each field were quantified from images captured at the initial time point. The proportion of PI-positive cells was assessed by manually counting macrophages.

### 2.16 Western blotting

Cells were lysed using a mixture of RIPA buffer (P0013B, Beyotime), PMSF (ST506, Beyotime), and phosphatase inhibitor cocktail (P1081, Beyotime) prepared at a ratio of 100:1:2 (v/v/v). The total protein was isolated and quantified using the BCA assay (EC0001, Sparkjade). The proteins were then separated by SDS-PAGE, transferred to PVDF membranes (IPVH00010, Merck Millipore), and blocked with 5% (w/v) fat-free milk for 2 h. After washing with TBST, membranes were incubated overnight at 4°C with diluted primary antibodies, followed by a 1-h incubation at room temperature with HRP-conjugated secondary antibodies. Protein bands were visualized using the ECL chemiluminescent substrate and imaged with a Gel Imaging Analysis System (Tianneng, Shanghai, China). All antibody data (**Supplementary Table S2**) and original gel blots are presented in the supplementary file.

### 2.17 Statistical analysis

All experiments were carried out in triplicate and repeated in at least three biological replicates. Data are presented as mean values ± standard deviations. Statistical analysis was performed using SPSS v.26.0 (IBM, Chicago, IL, USA). Group differences were evaluated by one-way analysis of variance (ANOVA) followed by Tukey’s *post hoc* test for parametric data. Non-parametric data were analyzed using the Kruskal-Wallis test with Dunn’s multiple comparisons correction. A *P*-value < 0.05 was considered statistically significant.

## 3 Results

### 3.1 CIN inhibits *C. albicans* proliferation and disrupts cell wall integrity

**Figure 1:**
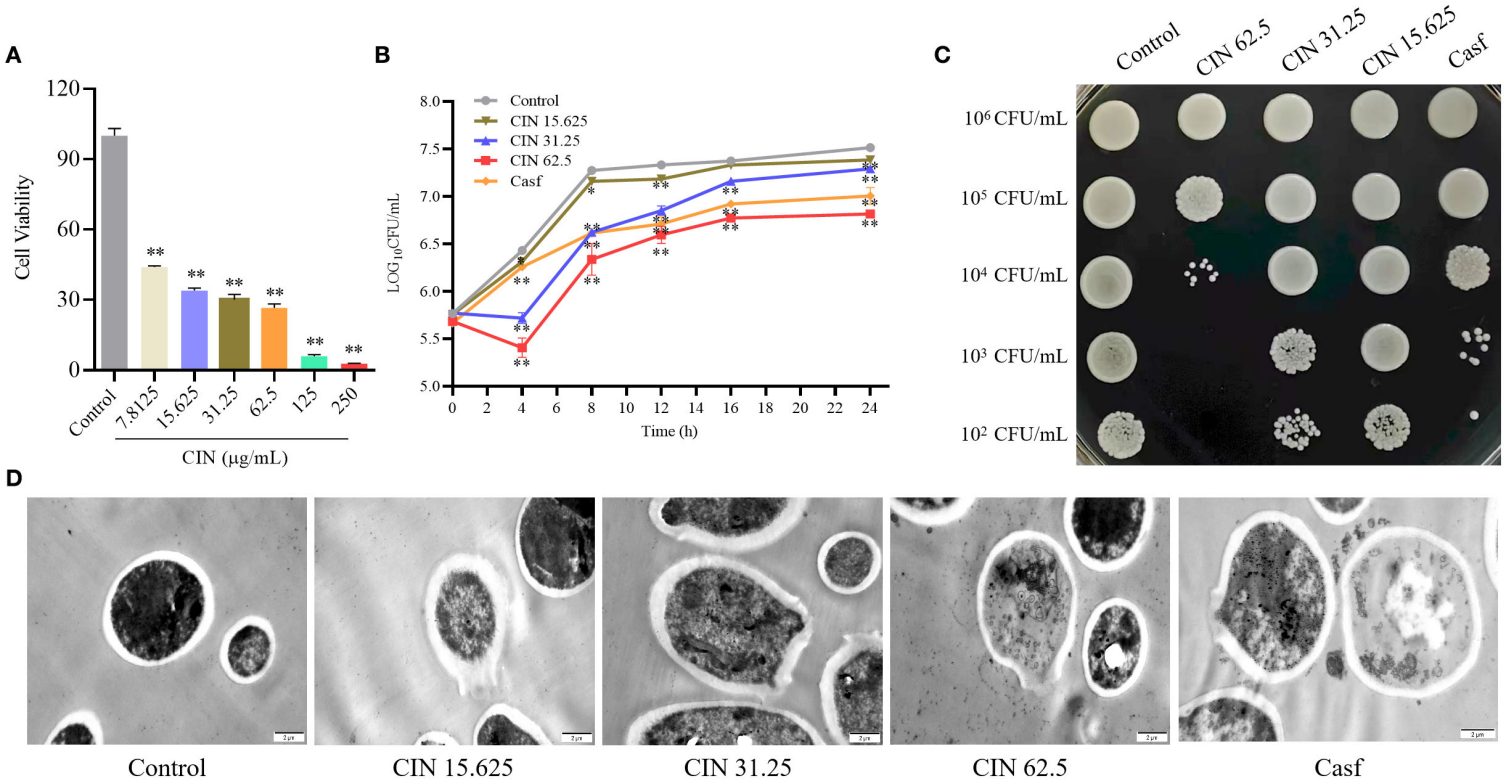
Antifungal activity of CIN against *C*. *albicans.* **(A)** Cell viability of *C*. *albicans* after 24 h exposure to CIN (0-250 μg/mL), assessed by CCK-8 assay. **(B)** Time-growth curve of *C*. *albicans* treated with CIN versus the untreated control. **(C)** Inhibitory effect of CIN on *C*. *albicans* colony growth on YPD plates. **(D)** Representative TEM images of *C*. *albicans*; scale bars: 2 μm. Significant differences from the control group are indicated as * *P* < 0.05, ** *P* < 0.01.

Broth microdilution assays determined a CIN MIC of 31.25 μg/mL against *C. albicans* (**Supplementary Figure S2**). Assessment of cell viability using the CCK-8 assay demonstrated concentration-dependent inhibition of *C. albicans* SC5314 growth (>7.8125 μg/mL), with near-complete metabolic suppression at 250 μg/mL (**Figure 1A**). SYTO9/PI double staining showed a significant increase in red fluorescence (dead cells) with rising CIN concentrations, confirming its inhibitory and fungicidal effects on *C. albicans* proliferation (**Supplementary Figure S3**). Time-growth curves revealed concentration- and time-dependent antifungal activity, showing significantly decreased log_10_CFU values in CIN-treated groups compared to controls (**Figure 1B**). CIN treatment also markedly impaired colony formation capacity (**Figure 1C**). TEM imaging demonstrated dose-dependent ultrastructural damage, including cell wall thinning, disintegration, cytoplasmic leakage, and uneven electron density (**Figure 1D**). Collectively, CIN inhibits *C. albicans* proliferation and compromises cell wall integrity.

### 3.2 CIN induces cell wall remodeling in *C. albicans*

**Figure 2:**
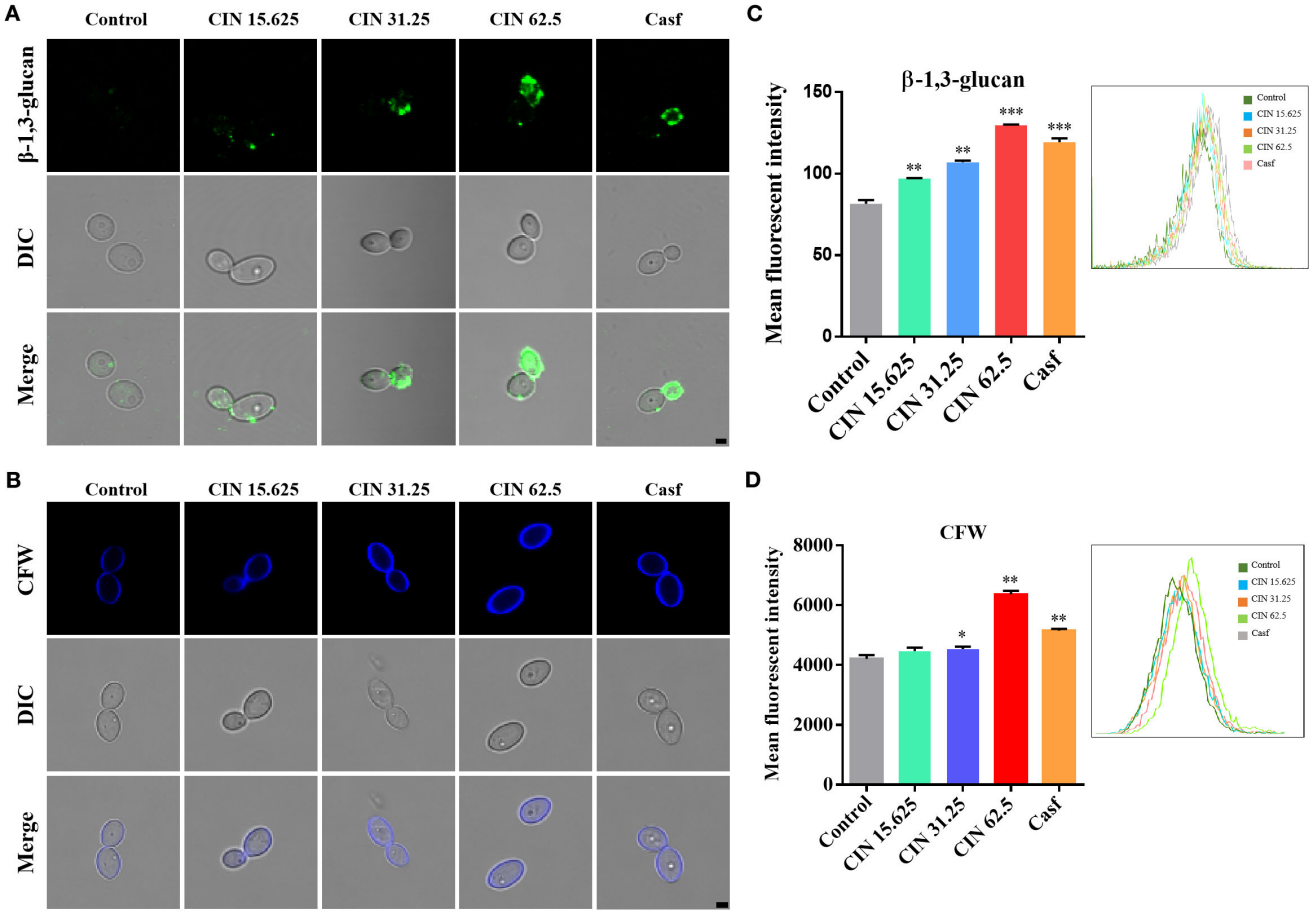
Surface exposure of β-1,3-glucan and chitin in *C*. *albicans*. **(A)** Representative immunofluorescence images of β-1,3-glucan exposure, stained with mouse anti-β-1,3-glucan antibody followed by Cy3-conjugated goat anti-mouse IgG (green). **(B)** Chitin staining with calcofluor white (CFW; blue). scale bar: 2 µm. Mean fluorescence intensity of **(C)** β-1,3-glucan and **(D)** total chitin was quantified by flow cytometry. Caspofungin (Casf., 0.0039 μg/mL) was set as the positive control. Significant differences compared with the control group are indicated as *P < 0.05, **P < 0.01 and ***P < 0.001.

The impact of CIN on the cell wall structure of *C. albicans* was investigated using fluorescence microscopy and flow cytometry. After 12 h of exposure to CIN, exposure of β-1,3-glucan was observed in a concentration-dependent manner. The mean fluorescence intensity (MFI) in the 31.25 μg/mL and 62.5 μg/mL CIN groups was significantly higher than in the control group (**Figures 2A, C**). Furthermore, CIN treatment increased chitin deposition, as indicated by an increase in calcofluor white (CFW) fluorescence compared with the control (**Figures 2B, D**). These results demonstrate that CIN remodels the *C. albicans* cell wall by increasing surface exposure of β-1,3-glucan and promoting chitin deposition.

### 3.3 Transcriptomic profiling of CIN-treated *C. albicans*

**Figure 3:**
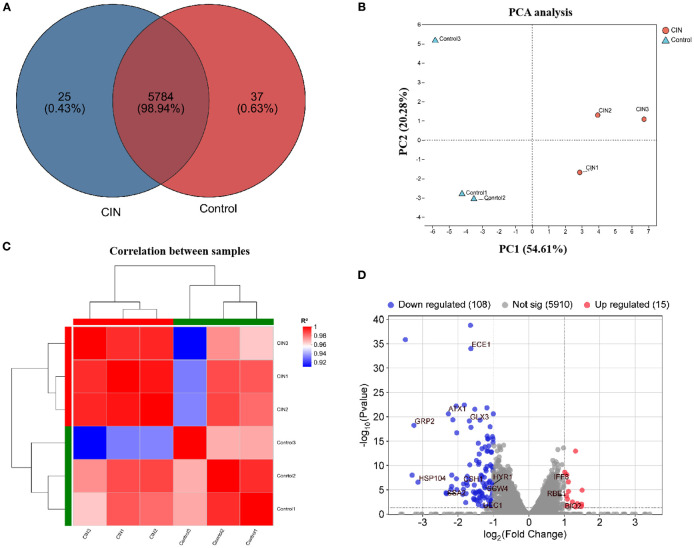
Transcriptomic profiling of *C*. *albicans* under CIN treatment. **(A)** Venn diagram showing transcript counts in CIN-treated versus untreated samples. **(B)** PCA plot illustrating sample correlation and reproducibility. **(C)** Hierarchical clustering heat map of gene expression; red indicates high correlation and blue indicates low correlation. **(D)** Volcano plot of DEGs with |log_2_FC| > 1 and FDR < 0.05; red dots indicate up-regulated and blue dots indicate down-regulated genes.

To investigate the mechanism of action of CIN, we performed transcriptomic analysis of CIN-treated cells. *C. albicans* SC5314 cells were cultured for 24 h with or without CIN (31.25 μg/mL) prior to total RNA extraction. RNA sequencing of three biological replicates of each group generated 39.25 Gb of clean data (≥6.13 Gb per sample), with Q30 scores > 96.11% (**Supplementary Table S3**). Reference genome alignment achieved mapping rates ranging from 97.01 to 97.27%, yielding 5,846 expressed transcripts (**Supplementary Table S4**). Comparative analysis identified 5,784 shared transcripts, with 37 exclusively expressed in CIN-treated cells and 25 unique to controls (**Figure 3A**). Principal component analysis (PCA) and hierarchical clustering revealed that samples from different groups formed distinct clusters, whereas those from the same group clustered closely together, indicating strong intra-group correlation and unique transcriptome profiles (**Figures 3B, C**). CIN significantly impacted the *C. albica*ns transcriptome, resulting in 123 differentially expressed genes (DEGs): 15 upregulated and 108 downregulated (**Figure 3D**). Among the down-regulated genes, notable examples include *ECE1, GPR2, HSP104, SCW4*, and *HYR1*, which encode key regulators of hyphal growth, cell wall biogenesis, and biofilm formation.

### 3.4 Functional annotation and enrichment of DEGs

**Figure 4:**
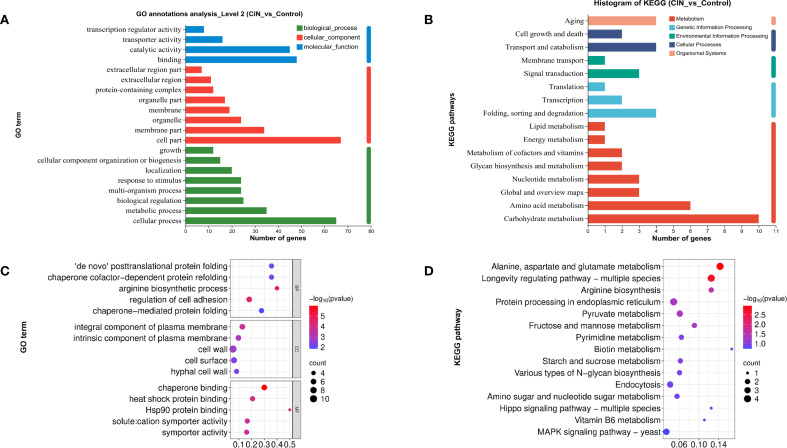
Functional annotation and enrichment analysis of DEGs in *C*. *albicans* after CIN treatment. **(A)** GO and **(B)** KEGG function annotation; **(C)** GO and **(D)** KEGG pathway enrichment analyses of the DEGs.

GO analysis revealed significant alterations in cellular components, particularly in cell membrane parts, and in molecular functions including binding and catalytic activities (**Figure 4A**). These findings were consistent with the results of the functional annotation clustering analysis (**Supplementary Table S5**). Separate GO analysis of the 15 up-regulated genes produced no statistically significant enrichments, consistent with the primary response being dominated by repression of cell wall-related functions. KEGG annotation revealed that CIN significantly influenced carbohydrate and amino acid metabolism, with downstream effects on transport systems and on cellular processes that regulate cell growth and death (**Figure 4B**). GO enrichment further demonstrated that CIN mediated the disruption of protein folding, cell wall organization, hyphal cell wall biogenesis, and cell surface assembly (**Figure 4C**). KEGG pathway enrichment analysis showed that CIN-responsive DEGs were primarily linked to carbohydrate metabolism, amino acid biosynthesis, and the MAPK pathway (**Figure 4D**).

### 3.5 CIN alters expression of cell wall remodeling genes in *C. albicans*

**Figure 5:**
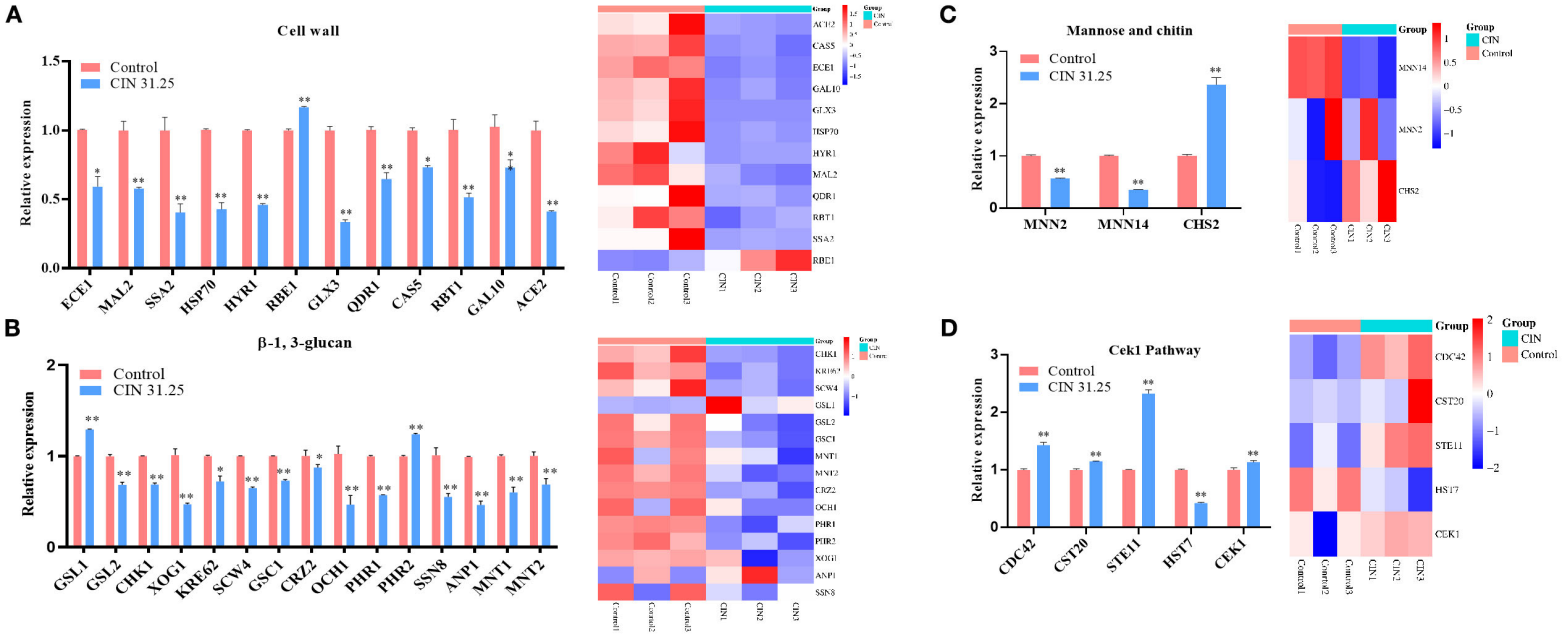
Expression validation of DEGs identified by transcriptome analysis. RT-qPCR was performed to validate DEGs related to the synthesis and degradation of **(A)** cell wall components, **(B)** β-1,3-glucan, **(C)** mannose and chitin, and **(D)** genes involved in the Cek1 pathway (left panels). Corresponding transcriptome heatmaps are shown (right panels). Statistical comparisons with the control group are indicated as *P < 0.05, **P < 0.01.

To validate the transcriptomic findings, we performed RT-qPCR analysis on 35 cell wall-related DEGs identified through functional annotation clustering. The qPCR results revealed that CIN markedly down-regulated 11 cell wall genes, including integrity regulators (*ECE1*, *HYR1*, *RBT1*), stress response mediators (*HSP70*, *GLX3*), ion transporters (*QDR1*, *MAL2*), and morphogenesis factors (*SSA2*, *CAS5*, *GAL10*, *ACE2*) (**Figure 5A**). CIN also suppressed 13 β-glucan biosynthesis-related genes, including structural components (*GSL2, PHR1, XOG1*), signaling elements (*CRZ2, CHK1*), glycosyltransferases (*OCH1*, *MNT1*, *MNT2*, *GSC1*), and regulatory factors (*KRE62, SCW4, SSN8, ANP1*) (**Figure 5B**). In addition, CIN significantly decreased the expression of key mannosyltransferase genes (*MNN14, MNN2, ANP1*) but increased that of the chitin synthase gene *CHS2* (**Figures 5B, C**), triggering excessive chitin deposition in the inner cell wall layer (**Figure 2**). Notably, CIN activated the Cek1 MAPK signaling pathway through up-regulation of *CEK1, CDC42, CST20*, and *STE11* (**Figure 5D**).

### 3.6 Molecular docking analysis

Molecular docking suggested potential binding of CIN to fungal cell wall targets (53.12% had binding energies of ≤-5.0 kcal/mol), including cell wall-associated proteins, β-glucan regulators, and Cek1 pathway components (**Supplementary Table S6**). Further, representative binding modes indicated hydrophobic interactions and hydrogen bonds constitute the primary binding mechanisms (**Supplementary Figure S4**). These *in silico* analyses suggest potential interaction sites between CIN and fungal proteins, although functional validation, such as suppressor mutation mapping, remains essential for mechanistic confirmation.

### 3.7 CIN enhances macrophage phagocytosis and clearance of *C. albicans*

**Figure 6:**
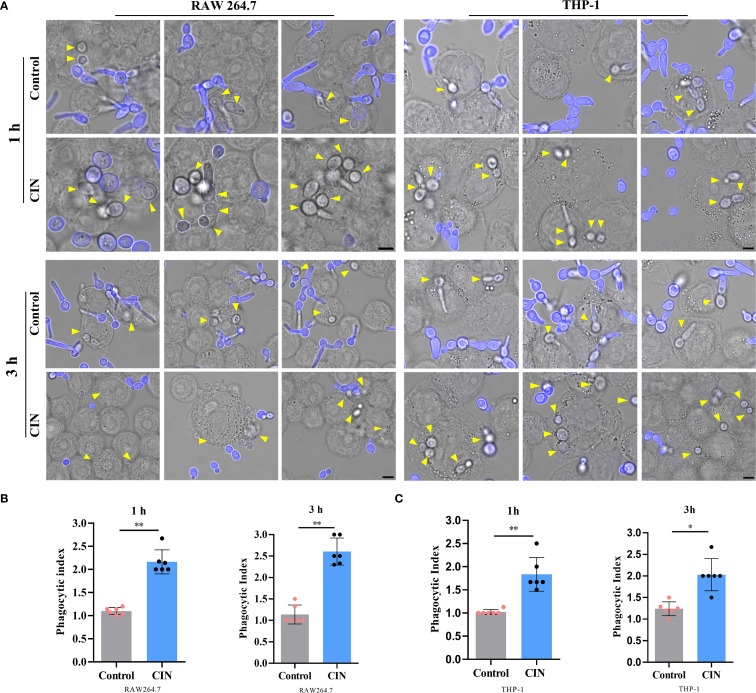
CIN enhances *C*. *albicans* phagocytosis by macrophages. **(A)** Representative fluorescent images of engulfed *C*. *albicans* (scale bar: 5 μm). RAW264.7 and THP-1-derived macrophages were incubated with *C*. *albicans* pretreated with CIN (31.25 μg/mL, 12 h) at an MOI of 3 for 1 and 3 h, respectively. External *C*. *albicans* cells were stained blue with CFW, while phagocytic cells remained unstained (yellow arrows). The phagocytic indices of RAW264.7 **(B)** and THP-1 **(C)** were calculated. Significant differences in comparisons with the control group: *P < 0.05, **P < 0.01.

**Figure 7:**
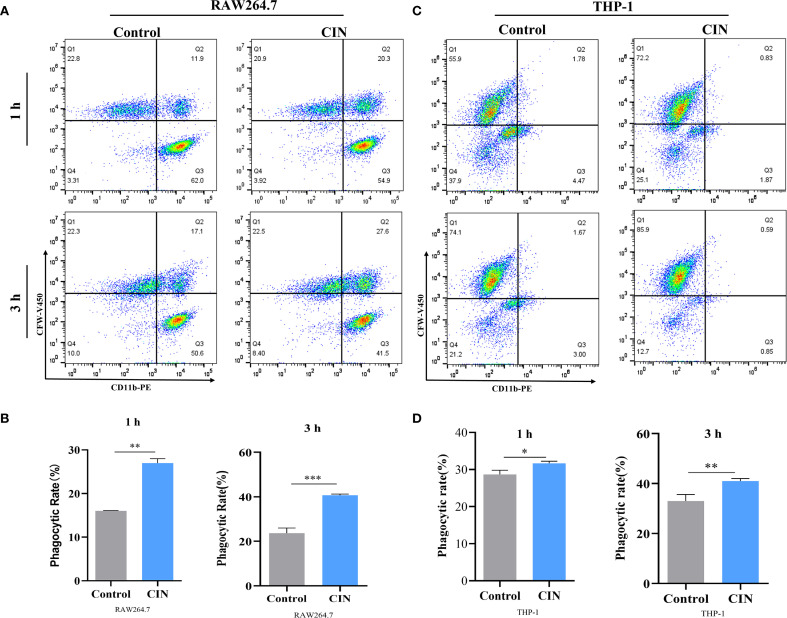
Phagocytic rates of RAW264.7 **(A, B)** and THP-1-derived macrophages **(C, D)** at 1 and 3 h are shown. Macrophages were stained with a PE-conjugated anti-CD11b antibody (X-axis). *C. albicans* SC5314 cells were pre-treated with or without CIN (31.25 μg/mL) for 12 h and then labeled with CFW-V450 (Y-axis) prior to co-culture. Phagocytosis was assessed by flow cytometry, and the phagocytic rate was defined as the percentage of CD11b^+^ CFW-V450^+^ cells within the live macrophage gate. Macrophages were incubated with *C. albicans* pretreated with CIN (31.25 μg/mL) for 12 h, and phagocytic rates were examined by FACS. Statistical significance versus the control group is denoted as *P < 0.05, **P < 0.01, ***P < 0.001.

To assess immune recognition, RAW264.7 and THP-1-derived macrophages were co-cultured with either CIN-pretreated or untreated *C. albicans*. Fluorescence microscopy revealed significantly enhanced phagocytosis of CIN-treated fungi, as demonstrated by increased phagocytic indices in both macrophage types at 1 h and 3 h post-infection (**Figures 6A-C**). Flow cytometry confirmed elevated phagocytosis frequencies, with significantly higher rates in the CIN-treated groups than in the controls at both time points (**Figures 7A-D**).

### 3.8 CIN enhances macrophage-mediated clearance of *C. albicans* and immune response

**Figure 8:**
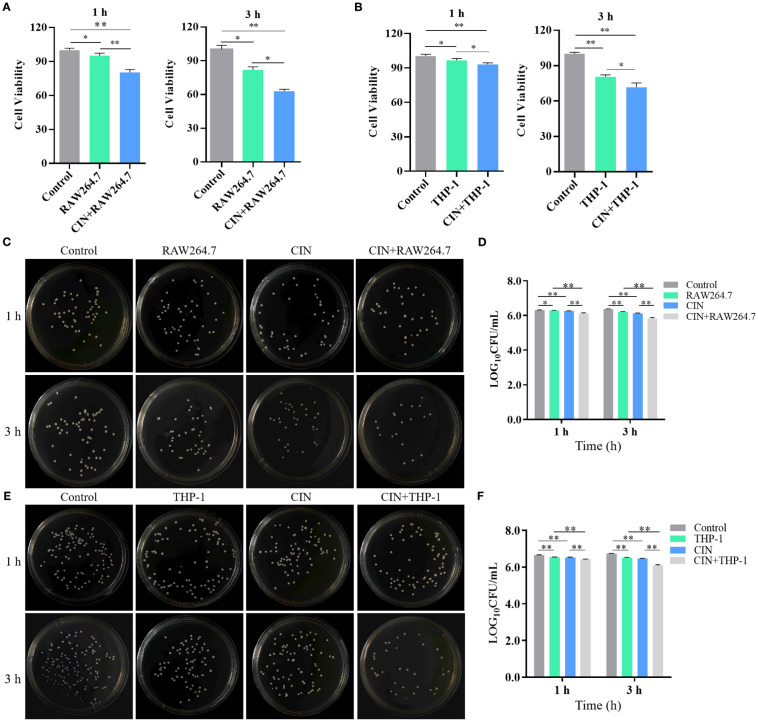
CIN enhances macrophage-mediated clearance of *C*. *albicans*. Survival of *C*. *albicans* following 1 or 3 h co-culture with **(A)** RAW264.7 and **(B)** THP-1-derived macrophages. *C*. *albicans* was pretreated with CIN (31.25 μg/mL) or vehicle for 12 h, then co-cultured with macrophages. Residual fungal metabolic activity was assessed via XTT reduction assays. Viable *C*. *albicans* CFUs were quantified after 1 or 3 h co-culture with **(C, D)** RAW264.7 and **(E-F)** THP-1 cells. Fungi were plated on YPD agar and incubated at 37 °C for 24 h. Statistical significance is indicated as *P < 0.05 and **P < 0.01.

**Figure 9:**
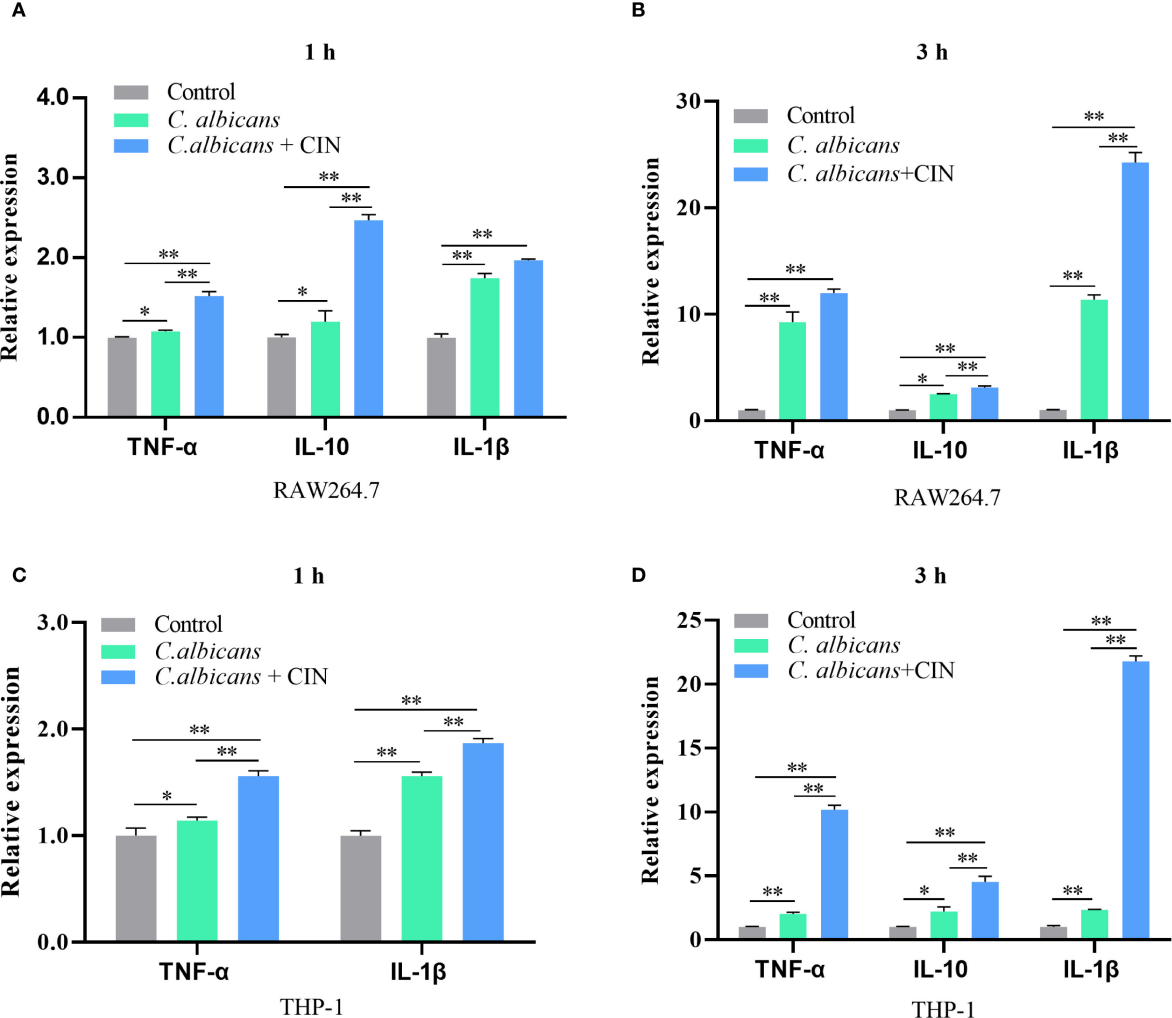
CIN modulates cytokine responses in macrophages during *C*. *albicans* infection. **(A, B)** TNF-α, IL-1β, and IL-10 mRNA expression in RAW264.7 cells and **(C, D)** THP-1 cells were measured after 1 or 3 h infection with *C*. *albicans* pretreated with CIN (31.25 μg/mL, 12 h) using RT-qPCR. Statistical significance is denoted as *P < 0.05 and **P < 0.01.

CIN-pretreated *C. albicans* exhibited a >60% increase in clearance by both RAW264.7 and THP-1-derived macrophages compared to the macrophage group (macrophages co-cultured with untreated *C. albicans*) or to the control group (*C. albicans* alone) (**Figures 8A, B**). CIN significantly enhanced the antifungal activity in RAW264.7 and THP-1 cells compared with the controls (**Figures 8C-F**). Colony-forming assays showed that the reduction in CFU in the co-culture system was significantly greater than the sum of the reductions caused by CIN alone and by macrophages alone (**Supplementary Table S7**). Consistent with enhanced phagocytic activity, the expression of proinflammatory cytokines TNF-α and IL-1β was significantly elevated in RAW264.7 and THP-1 macrophages after 1- and 3-hour co-culture with CIN pretreated *C. albicans*, while expression of the immunoregulatory IL-10 increased moderately (**Figure 9**).

### 3.9 CIN inhibits *C. albicans* escape from macrophages

**Figure 10:**
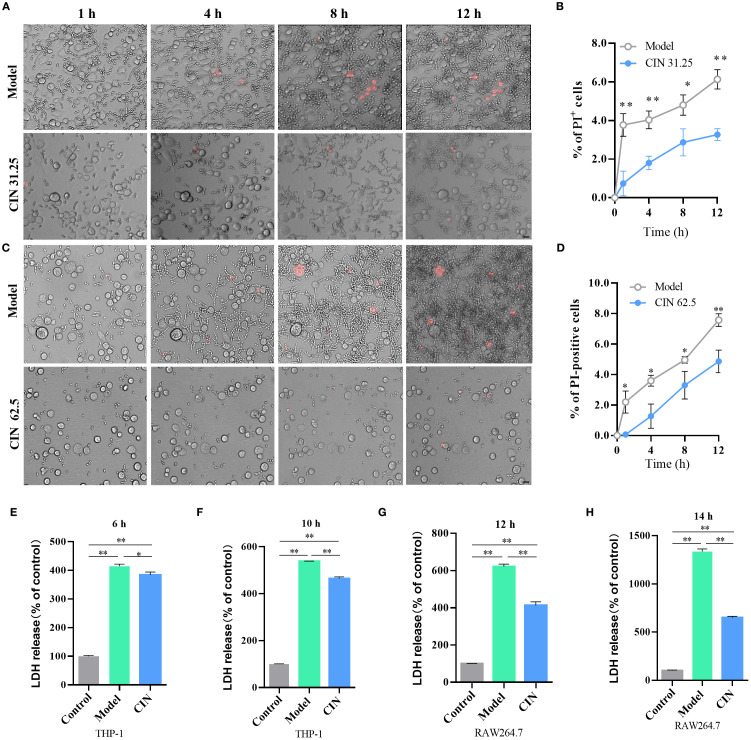
CIN attenuates *C. albicans* escape from macrophages. **(A, C)** Representative microscopy images of THP-1-derived macrophages showing *C. albicans* escape dynamics. Scale bar: 100 µm. **(B, D)** Quantification of PI-positive cells at each time point. **(E-H)** LDH release was measured at 6 and 10 h post-infection in THP-1 macrophages and at 12 and 14 h post-infection in RAW264.7 macrophages after challenge with *C. albicans* pretreated with CIN (31.25 μg/mL, 12 h). Control, uninfected macrophage; Model, macrophage co-cultured with untreated *C. albicans*; CIN: macrophage co-cultured with *C. albicans* pretreated with CIN (31.25 μg/mL, 12 h). Statistical significance is indicated as *P < 0.05 and **P < 0.01.

Macrophage cell death induced by *C. albicans* is pivotal for both the fungal pathogen and the host. *C. albicans* uses this mechanism to escape and spread, whereas macrophages counter by releasing cytokines to trigger immune responses. PI staining of live cells was conducted to evaluate loss of membrane integrity *in situ*. After infection with *C. albicans*, the proportion of PI-positive cells rose steadily, indicating cell rupture and release of intracellular contents. Infection with CIN-treated *C. albicans* caused less macrophage membrane damage, as indicated by fewer PI-positive macrophages compared with the untreated control (**Figures 10A-D**). Assessment of macrophage lysis, measured by lactate dehydrogenase (LDH) release, showed a time-dependent increase upon challenge with untreated *C. albicans*. CIN pretreatment significantly reduced LDH release relative to macrophages infected with untreated *C. albicans*, demonstrating that CIN suppresses *C. albicans*-induced macrophage death (**Figures 10E, F**).

### 3.10 CIN enhances macrophage antifungal response against *C. albicans* via Dectin-1 signaling

**Figure 11:**
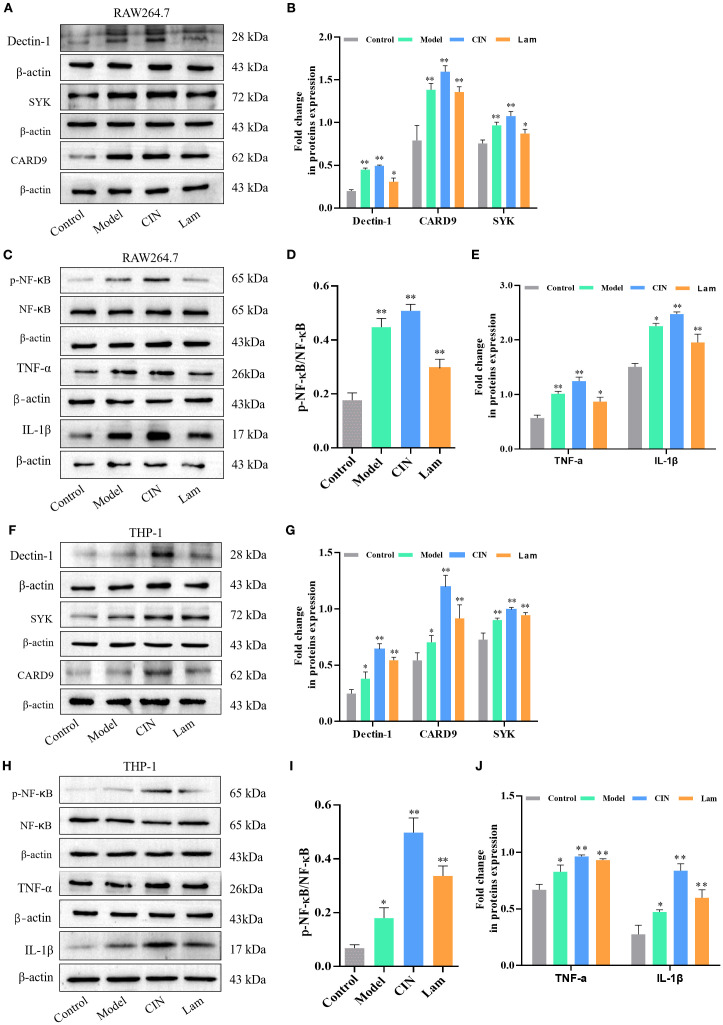
CIN amplifies macrophage activity against *C. albicans* through the Dectin-1 signaling pathway. **(A-E)** Dectin-1, SYK, CARD9, NF-κB, TNF-α and IL-1β protein levels in RAW264.7 and **(F–J)** THP-1 cells, were examined after 12 h infection with *C. albicans* pretreated with CIN (31.25 μg/mL, 12 h); macrophages were either pre-treated or not with laminarin (1.0 mg/mL, 30 min). Control, uninfected macrophages; Model, macrophages co-cultured with untreated C*. albicans*; CIN, macrophages co-cultured with *C. albicans* pretreated with CIN; Lam: macrophage pre-treated with laminarin and then co-cultured with *C. albicans* pretreated with CIN. Statistical comparisons with the control group are indicated as *P < 0.05, **P < 0.01.

Dectin-1 serves as the critical receptor for β-glucan recognition on immune cells. To investigate CIN’s immunomodulatory mechanism, we analyzed Dectin-1 signaling components (Dectin-1, SYK, and CARD9), NF-κB activation, and proinflammatory cytokines (TNF-α and IL-1β) in RAW264.7 and PMA-differentiated THP-1 macrophages during a 24-h fungal infection. CIN-pretreated *C. albicans* markedly enhanced Dectin-1 receptor expression, activated downstream signaling (SYK phosphorylation and CARD9 recruitment), and induced NF-κB phosphorylation in both THP-1 (**Figures 11A-E**) and RAW264.7 macrophages (**Figures 11F, G**). This cascade increased the secretion of TNF-α and IL-1β compared with the untreated *C. albicans*-infection group. Crucially, laminarin (a Dectin-1 inhibitor) abrogated these effects, confirming the critical role of Dectin-1 in CIN-mediated antifungal immunity.

## 4 Discussion

Research into drugs that modulate the host immune response to fungal pathogens is crucial for developing new antifungal therapies. This approach not only targets the pathogen directly but also enhances the host’s capacity to combat infections, potentially leading to more effective treatment strategies. This study demonstrates that CIN triggers exposure of β-glucan and chitin on the *C. albicans* cell wall, thereby promoting phagocytosis while attenuating macrophage cell death. To our knowledge, this is the first report to demonstrate CIN’s mode of action in augmenting macrophage-mediated clearance of *C. albicans* through enhanced phagocytosis and pro-inflammatory cytokine production.

Previous studies have shown that CIN effectively kills *Candida* species by disrupting the cell structure and altering metabolic processes, leading to cell death (Chen et al., 2019a; Huang et al., 2019). The present study shows that the MIC of CIN against *C. albicans* SC5314 is 31.25 μg/mL, aligning with previous research findings (Taguchi et al., 2013). Time-growth curves reveal that CIN significantly inhibits *C. albicans* growth within the first 12 h. TEM assays show that CIN compromises the integrity of the *C. albicans* cell wall and exposes β-glucan. We previously reported that antifungal agents, such as CIN and sodium houttuyfonate unmask the inner β-glucan (Ma et al., 2020, 2021; Wang et al., 2024). These structural modifications coincide with increased chitin content in CIN-treated cultures, a phenomenon also observed after exposure to micafungin and caspofungin (Guirao-Abad et al., 2018; Wagner et al., 2023).

Beta-glucan and chitin are recognized as key PAMPs that activate immune responses via interaction with pattern-recognition receptors (PRRs) on immune cells. The molecular mechanisms by which CIN modifies the structure of the fungal cell wall to enhance β-glucan exposure remain unexplored. Transcriptome sequencing identified 123 DEGs in *C. albicans* following CIN treatment. It is important to note that our transcriptomic analysis reflects the state of *C. albicans* after 24 h of CIN treatment, capturing adaptive responses alongside primary effects. Future studies using acute time points will be crucial to identify the direct molecular targets of CIN. Functional annotation clustering indicated that these genes were associated with the extracellular region, hyphal cell wall and cell surface. RT-qPCR confirmed that CIN down-regulated genes responsible for β-glucan masking. These genes orchestrate sophisticated mechanisms to conceal the immunogenic β-1,3-glucan. CHK1 facilitates β-glucan sequestration within the inner cell wall matrix, thereby evading phagocyte detection (Klippel et al., 2010). XOG1 enzymatically trims exposed β-1,3-glucan chains via exo-β-1,3-glucanase activity (De Assis et al., 2022). PHR2 remodels β-glucan crosslinks that are critical for structural integrity (Popolo et al., 2017). Intriguingly, GSL1 and PHR2 exhibited paradoxical up-regulation post-CIN treatment, which is discordant with transcriptomic trends. This up-regulation discrepancy may reflect post-transcriptional regulation or compensatory feedback loops, necessitating proteomic validation. Concurrently, CIN suppressed mannosyltransferases (MNN2, MNN14) essential for outer mannan layer biosynthesis, destabilizing the fungal cell wall and promoting β-glucan exposure. Our data demonstrate that CIN-induced transcriptomic alterations, specifically downregulation of β-glucan biosynthesis genes (e.g., *GSL2*, *PHR1*) and upregulation of chitin synthase (e.g., *CHS2*), collectively drive β-glucan unmasking through two synergistic mechanisms: 1) Reduced β-glucan deposition thins the inner wall layer, directly exposing concealed epitopes due to impaired *de novo* biosynthesis (Da et al., 2019; Degani and Popolo, 2019). 2) Compensatory chitin overproduction disrupts wall architecture, where *CHS2*-mediated chitin fibrils displace glucan chains and create spatial gaps, enhancing accessibility to immune receptors (Wheeler, 2023).

Molecular docking revealed high-affinity interactions between CIN and Cek1 pathway components, cell wall-associated proteins, and β-glucan regulators. Notably, the Cek1 MAPK pathway, recognized as a regulator of β-glucan exposure (Wagner et al., 2022), exhibited activation patterns. Specifically, the upstream components (CDC42, CST20, STE11, and CEK1) were up-regulated, while the downstream effector HST7 was slightly suppressed. This aligns with literature reports in which Cek1 activation correlates with β-glucan unmasking (Chen et al., 2019b, 2022; Wagner et al., 2022). CIN likely undermines *C. albicans* cell wall integrity by disrupting fungal β-glucan masking, obstructing mannan-dependent immune evasion, and altering Cek1-mediated stress adaptation.

Cell wall structural polysaccharides are vital in mediating interactions between fungi and the host immune system, initiating innate immune responses that are crucial for defending against fungal infections. Treatment with antifungal agents such as caspofungin can induce β-glucan unmasking, thereby enhancing β-glucan recognition by host immune cells (Wagner et al., 2023; Van Boerdonk et al., 2024). Our findings show that CIN markedly increases the phagocytic rate in both RAW264.7 and THP-1 cells. Furthermore, these increases in phagocytosis enhance macrophage-mediated inhibition of fungal propagation and *C. albicans* clearance, alongside elevated cytokine levels that promote pathogen elimination.

**Figure 12:**
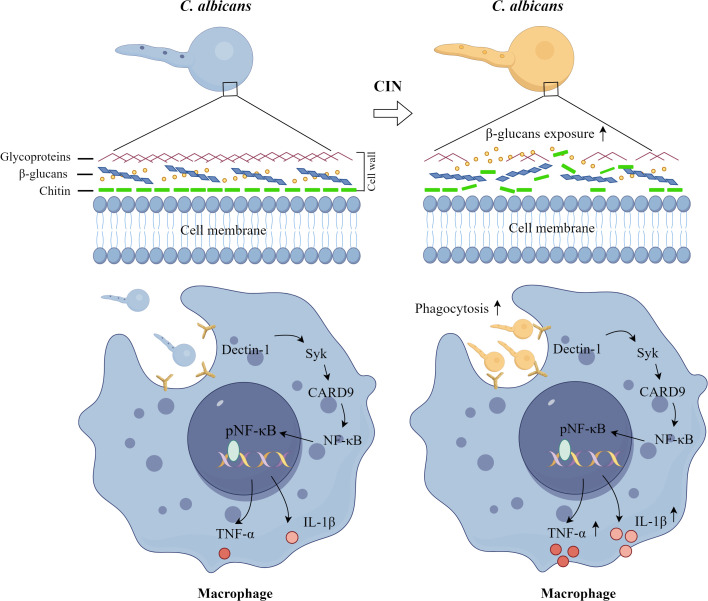
Proposed mechanism by which CIN enhances macrophage immunity against *C. albicans*. CIN promotes fungal detection and destruction through two mechanisms: it increases macrophage phagocytosis of *C. albicans* and activates the Dectin-1 signaling pathway.

The immune escape mechanism of *C. albicans* in macrophages is a multi-level process that involves morphological transformation, cell wall component regulation, and modulation of host signaling pathways. *C. albicans* evades macrophage recognition and phagocytosis by regulating its cell wall components or masking β-glucans (Hameed et al., 2021; Horton et al., 2021). Conversely, once engulfed, *C. albicans* undergoes morphological transformation into hyphae, enabling it to breach the constraints imposed by immune cells and escape (Wilson and Lorenz, 2023). *C. albicans* employs a dual inflammasome strategy to subvert host immunity: Gasdermin D (GSDMD)-mediated pyroptosis facilitates fungal escape via host cell lysis, whereas GSDMD-independent IL-1β production paradoxically enhances antifungal defenses through neutrophil recruitment and Th17 polarization (Ding et al., 2021). Our study demonstrated that CIN disrupts fungal immune evasion strategies through two synergistic mechanisms (**Figure 12**). First, CIN enhances fungal recognition and phagosomal killing, significantly increasing macrophages phagocytosis of *C. albicans*. This involves activation of the Dectin-1 signaling pathway, evidenced by increased expression of Dectin-1, SYK, and CARD9, enhanced NF-κB phosphorylation, and elevated secretion of TNF-α and IL-1β. Second, CIN inhibits fungal escape and subsequent macrophage lysis, as indicated by fewer PI-positive macrophage cells and reduced LDH release. Collectively, CIN exerts dual immunomodulatory effects by suppressing fungal stress adaptation while enhancing PRR-driven pathogen recognition and cytokine-mediated mycotoxicity.

In this study, we provide evidence that CIN modulates the cell wall composition of *C. albicans*, induces exposure of β-glucans, and enhances macrophage phagocytosis and clearance of *C. albicans*. Investigating these mechanisms will aid in creating new treatment strategies for *C. albicans* infections.

## Data Availability

The datasets presented in this study can be found in online repositories. The names of the repository/repositories and accession number(s) can be found in the article/**Supplementary Material**.
