# Supplementary material for: Cinnamaldehyde triggers cell wall remodeling and enhances macrophage-mediated phagocytic clearance of Candida albicans
Source: Front Cell Infect Microbiol. 2025 Sep 15;15:1647320. doi: 10.3389/fcimb.2025.1647320 (PMC12477181; doi:10.3389/fcimb.2025.1647320)
Supplement: Supplementary file 1 [file Table1.docx]

Supplementary Material

# Supplementary Tables

Table S1 RT-qPCR primers used in this study

| **Gene** | **Forward primer (5’ to 3’)** | | **Reverse primer (5’ to 3’)** | |  |
| --- | --- | --- | --- | --- | --- |
| *Candida albicans* SC5314 | | | | |  |
| *ACT1* | TGCTGAACGTATGCAAAAGG | | TGAACAATGGATGGACCAGA | |  |
| *GSC1* | ATCAACAACCACTTGCTTCG | | CCCATTCTCTAGGCACGA | |  |
| *GSL1* | TATGTCATTCAACTCGCCTTCCTTG | | AATCAGATATTGCGGCACCTAACG | |  |
| *GSL2* | GGCATCACTTCGCTCCCAAAC | | AGCTTGTTCTAACTTGTCGTTCTCC | |  |
| *PHR1* | GGTTTGGTTCTGGTTGATGG | | AGCAGCAGTTCCTGGACATT | |  |
| *PHR2* | CTCCTCCATTTCCAGAACCA | | CGTCTGAATCAACCTTGTCG | |  |
| *OCH1* | GCCGATTGGTACGCTAGAAGAATAC | | AATTCACGCAACATTGGATGACCTC | |  |
| *CHK1* | TGGTTCCGCAAGACGAGAATATGG | | TGTGCCAATCCCTTCCTCACTTTC | |  |
| *SSN8* | GTAGCACTAATACTGGGCGAAATGG | | GGCTTCAACAACTTCATCGAGATTG | |  |
| *CDC42* | CCGTCATTTCTCCTGCTTCGTTTG | | TTATTGGCACACCGGGACAATGG | |  |
| *CST20* | GCGTTTGGCGGTGAGAATAATGC | | GTGGAGGCGGCGGAGGTG | |  |
| *STE11* | CCTGCTCATGATCGCCATTCTAC | | ACCAAATTCTCCATCTTCCTCACC | |  |
| *HST7* | CAAGCGGTAGCCTAAGGAGTTCTG | | TAGGTGGCGGGCGTTGTCTC | |  |
| *CEK1* | GCTCAGGCTCAGGCTCAGG | | TGAATGAAACTTGACGAGGGGAAG | |  |
| *ANP1* | CTTTGGACAGTCGCCGTTAAACC | | CAGGAAGAGGACGCCAAACATTG | |  |
| *MNT1* | TGGCTTCTACCAGATCGAATGCTC | | GCGGCGGGTGATTGTGTTTG | |  |
| *MNT2* | CACCACCACAATCACCTTCATCAC | | ATTCTTCAGGCACCTTGACGATTTG | |  |
| *MNN2* | CAGACGCTTGGGCCAGAACTAC | | GAGTTTGTCTTTACCGCCAGCTTG | |  |
| *MNN14* | ATTGTGCAGGGTATACGTGGTGTG | | CCCCTCATCCATATCTCCCCAAGC | |  |
| *SSA2* | GCTTACGGTGCTGCTGTCCAAG | | GGAGCGACATCCAACAACAACAAG | |  |
| *HSP70* | AAGATGCCGTTGTTACCGTTCCAG | | ATAAGCAATGGCAGCAGCAGTAGG | |  |
| *HYR1* | GCTCAGGCTCAGGCTCACAATC | | TTGTCCAGAACCAGCTTCAGAACC | |  |
| *CHS2* | AGAGGAAGAAGAAGAGGAAGGTGAG | | GACTTCTTGTTGTGGAGGAGGTTC | |  |
| *ECE1* | GCCATCATCCACCATGCTCCAG | | CAGGAACAGTAGGTGCTTGGTCAG | |  |
| *MAL2* | AGAGGTTGAGCCGTGGATGAGAG | | CAATGAATGGTCGTCACCGTCCTG | |  |
| *RBE1* | AACTCCAGCTCCATCTTCGTCAAC | | GCGGTAGGTGTAGCGTCTGAATC | |  |
| *GLX3* | AGTTGCTGAAAAGGAAGGTGCTAC | | AGATTGTGGGTTGACACCAGTAAC | |  |
| *QDR1* | GGTTGGTGCAGCATTGATTTCAGG | | AAGTGACTCCTCCACCGATAGCC | |  |
| *CAS5* | GCCCGTTGTTGATGATGTCAATGG | | TCGCCACTTTCCCCTTCTTATTGC | |  |
| *RBT1* | TCTCCAGTTGCTCCAGGTGTCG | | AGCAGATGGTTGAATGGCAGGAAC | |  |
| *GAL10* | GCATGGTGGTGCAAATGGATTCG | | GCTCACCTGGGAACCCATCATTAC | |  |
| *ACE2* | ACAACAACAACAGCAACAGCAACC | | AATGTGGTTCTGGCGATGAGTTCC | |  |
| *KRE62* | ACCACGGCCAACAAGATGAAGATC | | TTGCCATTGCCATTGCCATTGC | |  |
| *XOG1* | GTCGCTGGTGAATGGTCTGCTG | | CCCTCATAACGTGCTCCTCTGTTG | |  |
| *CRZ1* | GGTGGTGGTGGTAGTAGTAACGATG | | TTGGATTGGTCGAAGTTCTTGATGC | |  |
| *SCW4* | CCAATTCACAACCACAAGCACCAG | | GGAGCAGCAGTAGCAGAAGTAACG | |  |
| *Mus musculus* | | | | |  |
| *TNF-α* | AAGCCTGTAGCCCACGTCGTA | | AAGGTACAACCCATCGGCTGG | |  |
| *IL-1β* | CAACCAACAAGTGATATTCTCCATG | | GATCCACACTCTCCAGCTGCA | |  |
| *IL-10* | CTTACTGACTGGCATGAGGATCA | | GCAGCTCTAGGAGCATGTGG | |  |
| **Gene** | | **Forward primer (5’ to 3’)** | | **Reverse primer (5’ to 3’)** | |
| *Actb* | | ATCTGGCACCACACCTTCTACAATG | | CACGCTCGGTCAGGATCTTCATG | |
| *Homo sapiens* | |  | |  | |
| *TNF-α* | | GGGGATTATGGCTCAGGGTC | | CGAGGCTCCAGTGAATTCGG | |
| *IL-1β* | | GCCAGTGAAATGATGGCTTATT | | AGGAGCACTTCATCTGTTTAGG | |
| *IL-10* | | ACCAAGACCCAGACATCA | | TTCACAGGGAAGAAATCG | |
| *GAPDH* | | ATGGAGAAGGCTGGGGCTC | | AAGTTGTCATGGATGACCTTG | |

**Table S2** Information of antibodies used in this study

| Antibody Name | Company | Cat No. | Dilution ratio |
| --- | --- | --- | --- |
| IL-1β | Abcam | EPR23851-127 | 1/1000 |
| TNF-a | Abcam | ab215188 | 1/1000 |
| Dectin-1 | BIOSS | bs-2455R | 1/500 |
| SYK | Zen-bio | 821293 | 1/500 |
| NF-κ B | AB Clonal | A19653 | 1/2000 |
| p-NF-κ B | AB Clonal | AP1294 | 1/1000 |
| CARD9 | Affinity | DF8387 | 1/1000 |
| β-actin | Zen-bio | 200068-8F10 | 1/1000 |
| Mouse secondary antibody | Zen-bio | 511103 | 1/10000 |
| Rabbit secondary antibody | Zen-bio | 511203 | 1/10000 |

**Table S3** Statistical results of sequencing data

| **Sample** | **Raw reads** | **Raw bases** | **Clean reads** | **Clean bases** | **Error rate(%)** | **Q20(%)** | **Q30(%)** | **GC content**  **(%)** |
| --- | --- | --- | --- | --- | --- | --- | --- | --- |
| CIN1 | 43853256 | 6621841656 | 43611064 | 6515334470 | 0.0119 | 98.82 | 96.30 | 37.56 |
| CIN2 | 44075728 | 6655434928 | 43833938 | 6539088791 | 0.0118 | 98.83 | 96.36 | 37.58 |
| CIN3 | 45869514 | 6926296614 | 45613476 | 6791617499 | 0.0118 | 98.86 | 96.45 | 37.72 |
| Control1 | 41204312 | 6221851112 | 40980922 | 6130965973 | 0.0118 | 98.86 | 96.45 | 37.42 |
| Conrtol2 | 43212326 | 6525061226 | 42967414 | 6423466489 | 0.012 | 98.76 | 96.11 | 37.70 |
| Control3 | 46185880 | 6974067880 | 45911950 | 6851657797 | 0.0119 | 98.79 | 96.20 | 37.49 |

**Table S4** Statistics of sequencing data comparison results

| **Sample** | **Total reads** | **Total mapped(%)** | **Multiple mapped(%)** | **Uniquely mapped (%)** |
| --- | --- | --- | --- | --- |
| CIN1 | 43611064 | 42388177 (97.20%) | 817979 (1.88%) | 41570198 (95.32%) |
| CIN2 | 43833938 | 42631824 (97.26%) | 841928 (1.92%) | 41789896 (95.34%) |
| CIN3 | 45613476 | 44345814 (97.22%) | 920338 (2.02%) | 43425476 (95.2%) |
| Control1 | 40980922 | 39755286 (97.01%) | 711983 (1.74%) | 39043303 (95.27%) |
| Conrtol2 | 42967414 | 41793603 (97.27%) | 810815 (1.89%) | 40982788 (95.38%) |
| Control3 | 45911950 | 44547176 (97.03%) | 760532 (1.66%) | 43786644 (95.37%) |

\

**Table S5** The functional annotation clustering analysis of DEGs

| **Term** | **Count** | **%** | **P Value** | **Fold Enrichment** | **FDR** |
| --- | --- | --- | --- | --- | --- |
| Extracellular region | 12 | 16.2162 | 0.0003 | 4.7318 | 0.0011 |
| Hyphal cell wall | 7 | 9.4595 | 0.0004 | 7.1441 | 0.0058 |
| Cell surface | 9 | 12.1622 | 0.0005 | 4.7318 | 0.0058 |
| Cell wall | 7 | 9.4595 | 0.0017 | 5.1625 | 0.0086 |
| Secreted | 8 | 10.8108 | 0.0017 | 4.3181 | 0.0086 |
| Fungal-type cell wall | 6 | 8.1081 | 0.0288 | 3.4131 | 0.1578 |
| Positive regulation of cell-substrate adhesion | 5 | 6.7568 | 0.0009 | 10.9946 | 0.0386 |
| Positive regulation of cell adhesion involved in single-species biofilm formation | 5 | 6.7568 | 0.0012 | 10.3283 | 0.0391 |
| C2H2-type 1 | 3 | 4.0541 | 0.0113 | 17.9201 | 0.3408 |
| C2H2-type 2 | 3 | 4.0541 | 0.0113 | 17.9201 | 0.3408 |
| Regulation of transcription by RNA polymerase II | 8 | 10.8108 | 0.0124 | 3.1162 | 0.2923 |
| Cell adhesion | 5 | 6.7568 | 0.0143 | 5.0417 | 0.1190 |
| RNA polymerase II cis-regulatory region sequence-specific DNA binding | 5 | 6.7568 | 0.0401 | 3.7846 | 0.6799 |
| N-linked (GlcNAc...) asparagine | 9 | 12.1622 | 0.0086 | 3.0146 | 0.3408 |
| Plasma membrane | 15 | 20.2703 | 0.0388 | 1.7408 | 0.1698 |
| MFS_dom | 7 | 9.4595 | 0.0003 | 7.4375 | 0.0565 |
| Major facilitator superfamily (MFS) profile | 7 | 9.4595 | 0.0004 | 6.9689 | 0.0506 |
| MFS_sugar_transport-like | 5 | 6.7568 | 0.0005 | 12.7500 | 0.0565 |
| MFS_trans_sf | 7 | 9.4595 | 0.0020 | 5.1990 | 0.1375 |
| **Term** | **Count** | **%** | **P Value** | **Fold Enrichment** | **FDR** |
| Arginine biosynthetic process | 4 | 5.4054 | 0.0002 | 30.2963 | 0.0188 |
| Arginine biosynthesis | 4 | 5.4054 | 0.0021 | 14.4281 | 0.0778 |
| Alanine, aspartate and glutamate metabolism | 4 | 5.4054 | 0.0059 | 10.1531 | 0.1073 |
| Stress response | 7 | 9.4595 | 0.0001 | 7.9872 | 0.0035 |
| Misfolded protein binding | 4 | 5.4054 | 0.0008 | 20.4952 | 0.0847 |
| Chaperone cofactor-dependent protein refolding | 4 | 5.4054 | 0.0024 | 14.3509 | 0.0650 |
| Longevity regulating pathway - multiple species | 4 | 5.4054 | 0.0087 | 8.8430 | 0.1073 |
| Cellular response to heat | 4 | 5.4054 | 0.0281 | 5.9275 | 0.3381 |

**Table S6** Molecualr docking analysis

**Table S7** Quantitative analysis of the synergistic effect between CIN and macrophages against *C. albicans*

| **Group** | Log_10_ CFU Reduction (Mean ± SD) | | |
| --- | --- | --- | --- |
|  | 1 h | | 3 h |
| CIN alone (in RPMI) | 0.05 ± 0.01 | | 0.25 ± 0.02 |
| RAW264.7 + Untreated Fungi | 0.03 ± 0.01 | | 0.16 ± 0.03 |
| RAW264.7+ CIN-treated Fungi | 0.20 ± 0.03** | | 0.54 ± 0.04* |
| Significantly greater than the sum of the CIN and RAW264.7 groups (***P* < 0.01, **P* < 0.05) | | | |
| **Group** | Log_10_ CFU Reduction (Mean ± SD) | | |
|  | 1 h | 3 h | |
| CIN alone (in RPMI) | 0.13 ± 0.01 | 0.26 ± 0.01 | |
| THP-1 + Untreated Fungi | 0.11 ± 0.01 | 0.23 ± 0.01 | |
| THP-1+ CIN-treated Fungi | 0.23 ± 0.02 ns | 0.66 ± 0.04** | |

Significantly greater than the sum of the CIN and THP-1groups (***P* < 0.01)

## Supplementary Figures


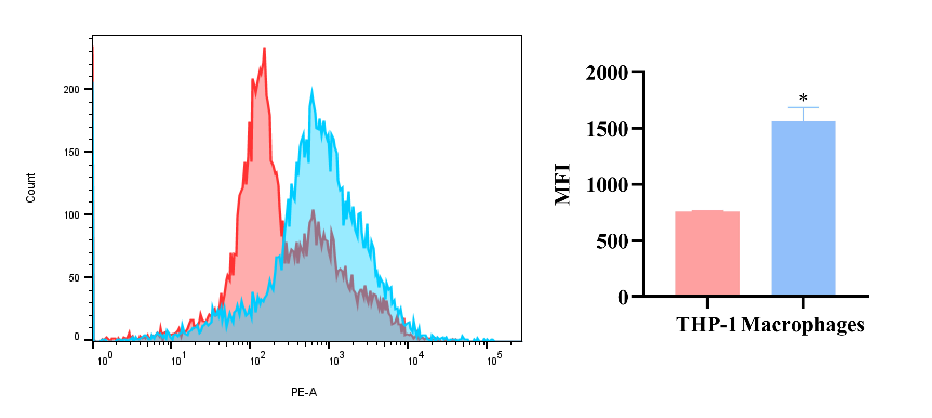


**Supplementary Figure S1.** Induction and identification of THP-1-derived macrophages

THP-1 cells were treated with 100 ng/mL phorbol 12-myristate 13-acetate (PMA) for 24 h. After harvesting, cells were incubated with PE Anti-human CD11b Antibody (E-AB-F1081D, Elabscience, China), and analyzed by BD CELFace™ flow cytometry using FlowJo software. Differences from the THP-1 group are considered significant at * p < 0.05.


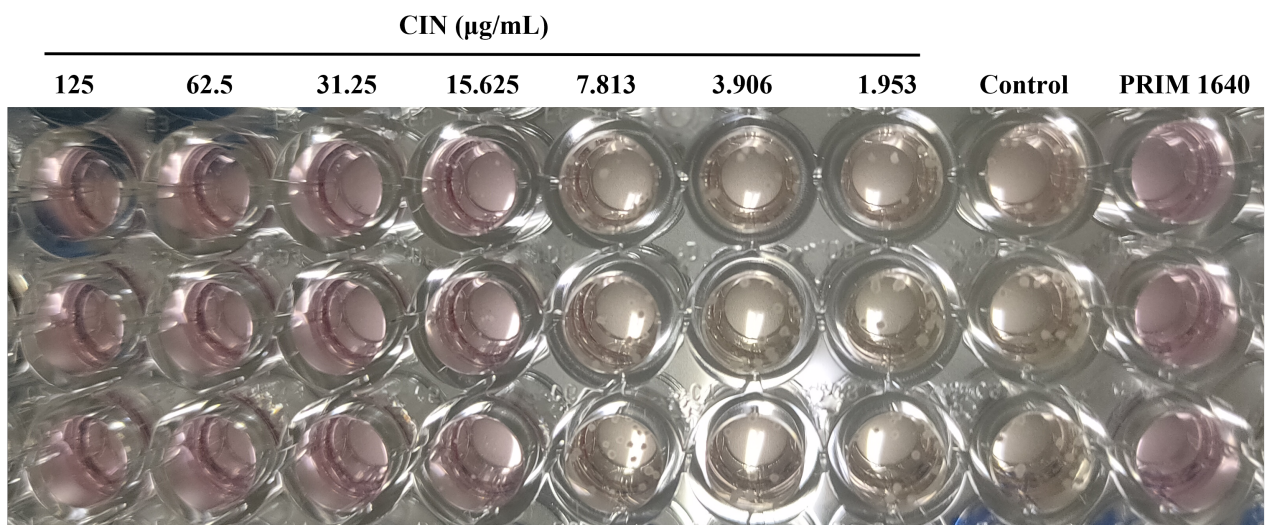


**Supplementary Figure S2.** Susceptibility assay of *C. albicans* SC5314 to cinnamaldehyde (CIN). The MIC of CIN against *C. albicans* was assessed using the broth dilution method in 96-well plates according to CLSI M27-A3 guidelines.


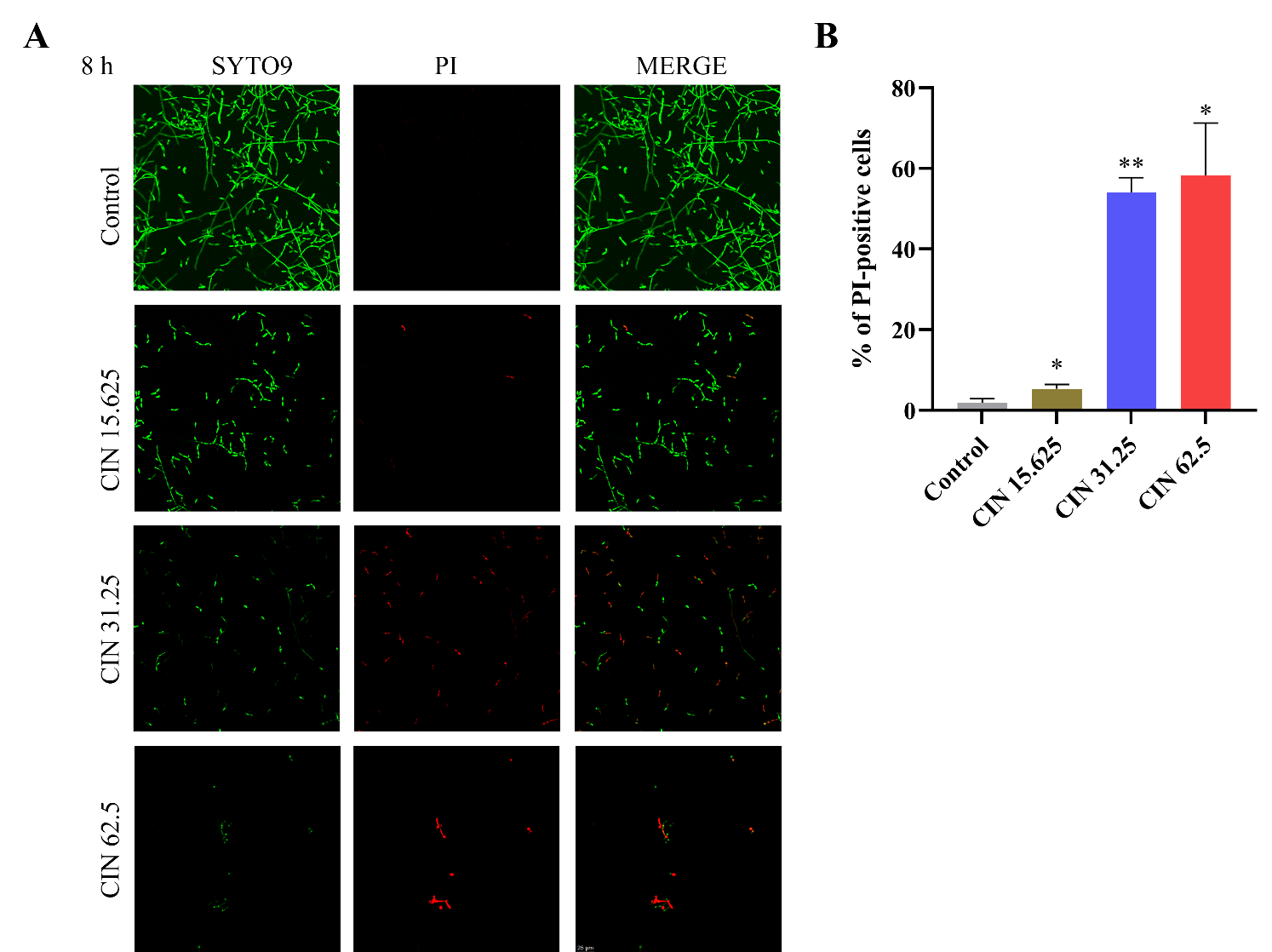


**Supplementary Figure S3. Evaluation of the effect of CIN on the cell viability of *Candida albicans* SC5314** (A) Representative fluorescence microscopy images of *C. albicans* SC5314 cells after treatment with the indicated concentrations of CIN for 8 h. Viable and dead cells were distinguished by SYTO9 (green, intact membranes) and propidium iodide (PI; red, compromised membranes) staining (MX4234-80T Kit, Shanghai Maokang Biotechnology). Scale bar: 20 μm.
(B) Quantitative analysis of cell death. The percentage of PI-positive cells was calculated from multiple fields of view and is presented as mean ± SD (n=3 independent experiments). ***P* < 0.01, **P* < 0.05 vs. untreated control (one-way ANOVA with Dunnett's post hoc test).


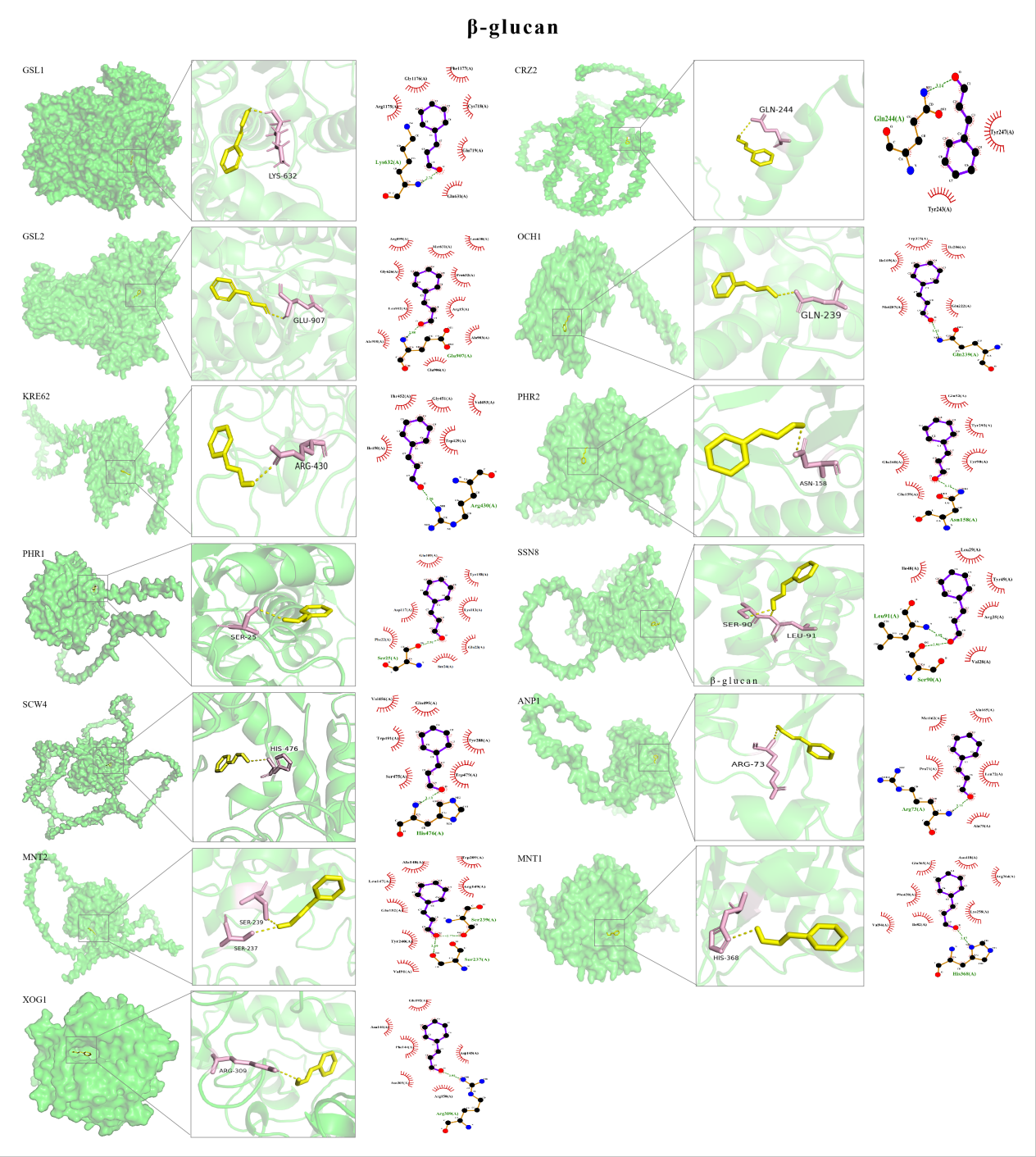


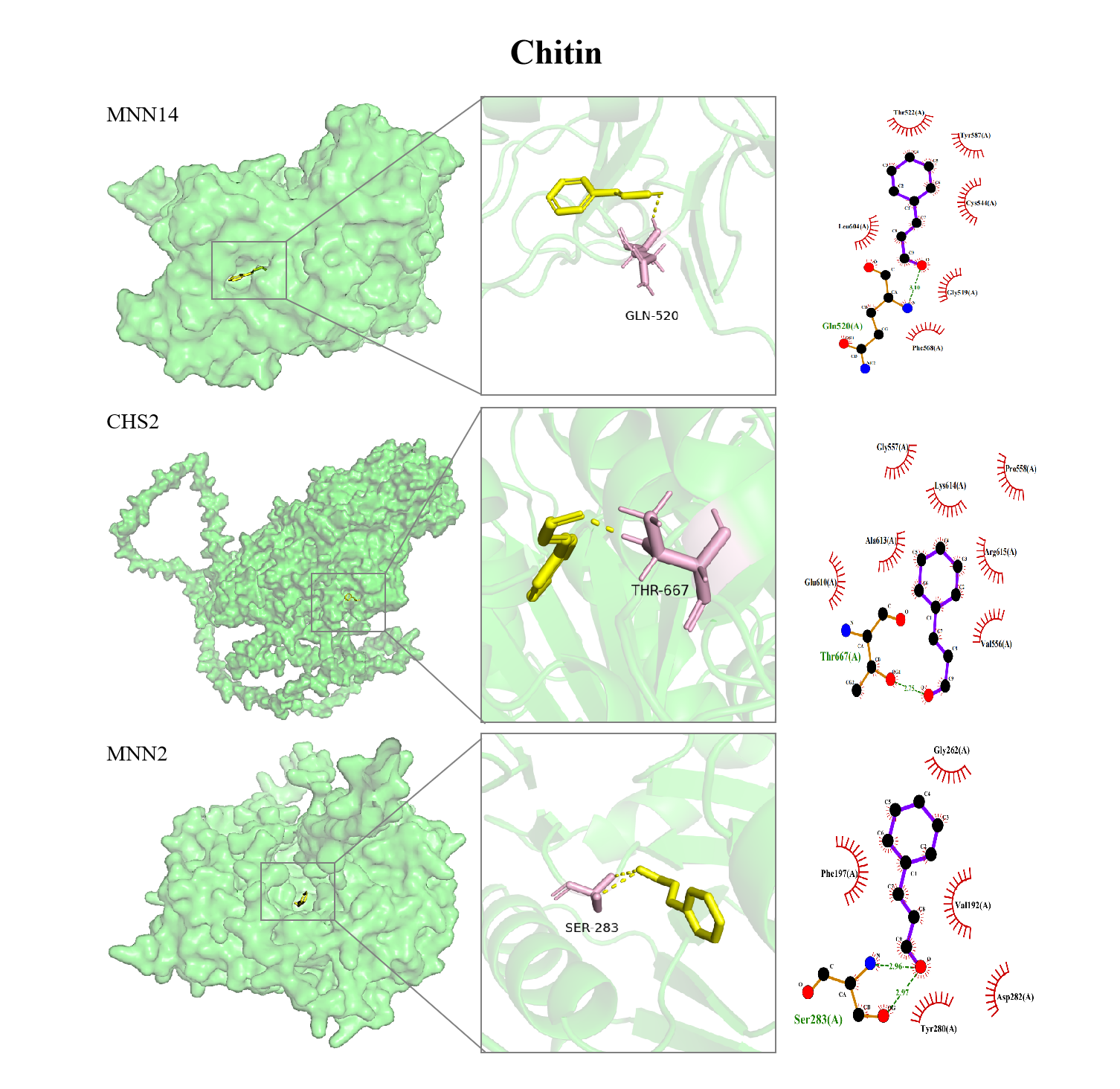


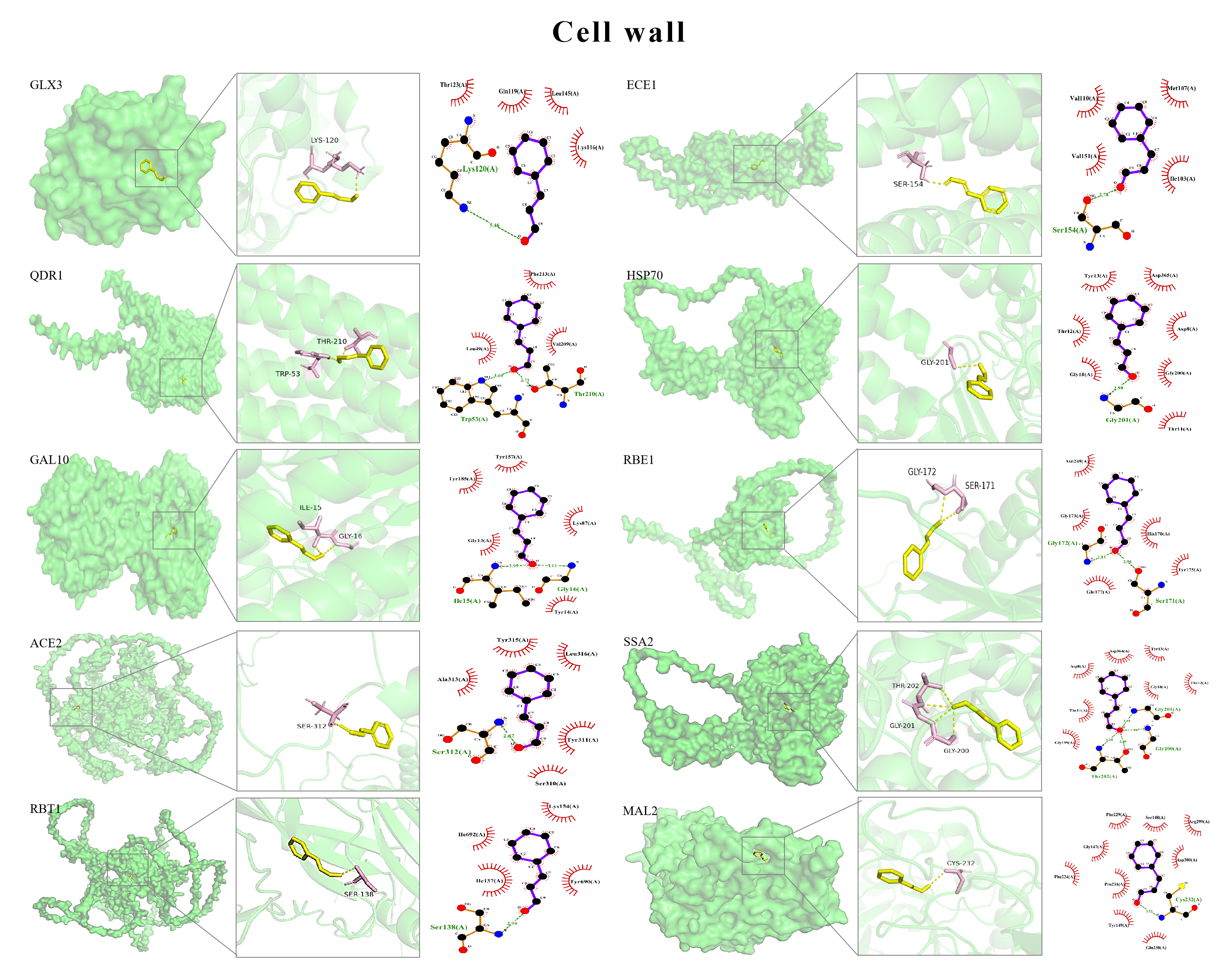


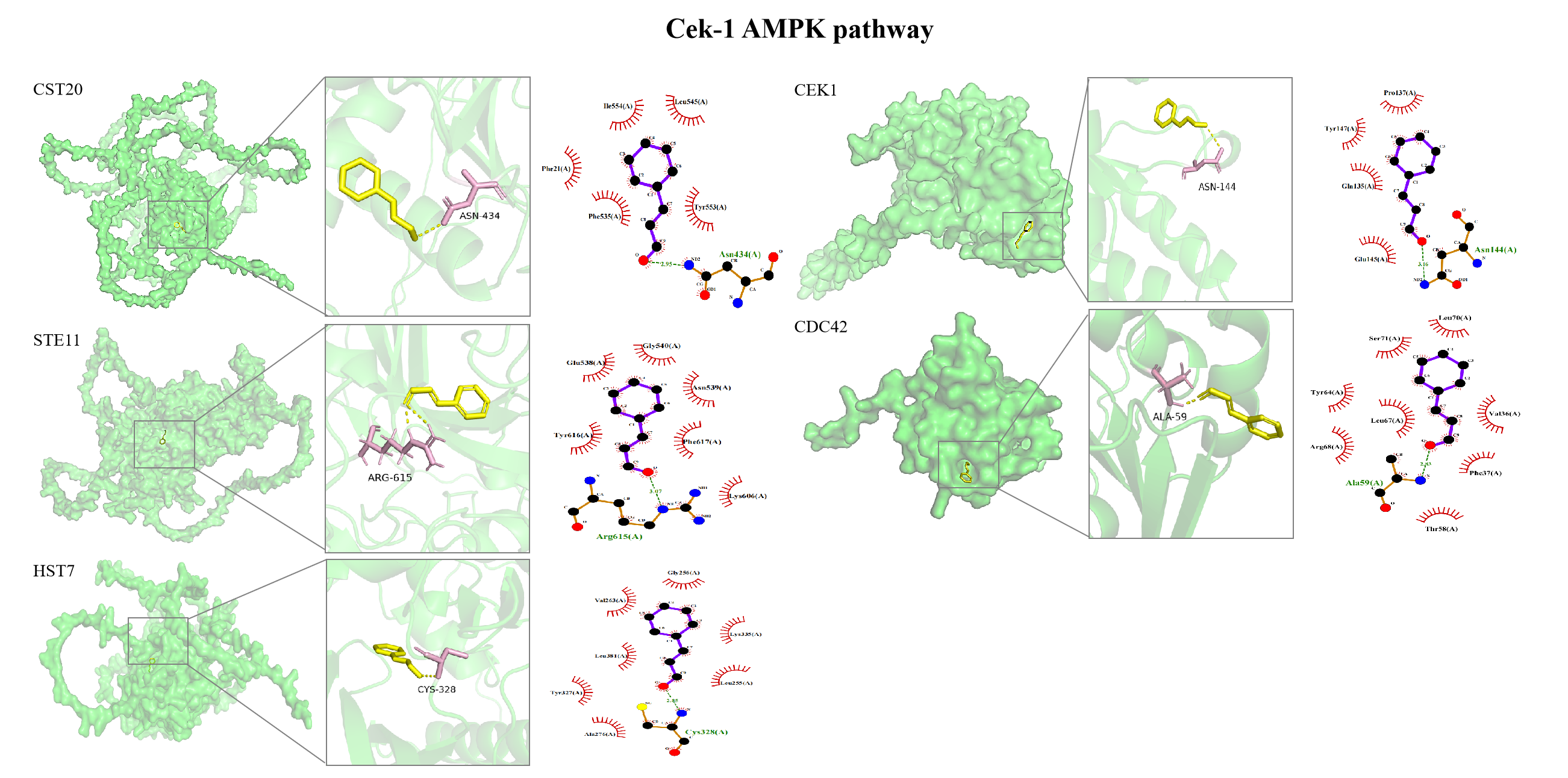


**Supplementary Figure S4**. Docking conformations of CIN with 13 β-glucan regulators, 3 chitin effectors,10 cell wall-associated proteins, and 5 components of CEK1 pathway.
